# Supplementary figures and images for: Downscaling livestock census data using multivariate predictive models: Sensitivity to modifiable areal unit problem
Source: PLoS One. 2020 Jan 27;15(1):e0221070. doi: 10.1371/journal.pone.0221070 (PMC6984718; doi:10.1371/journal.pone.0221070)

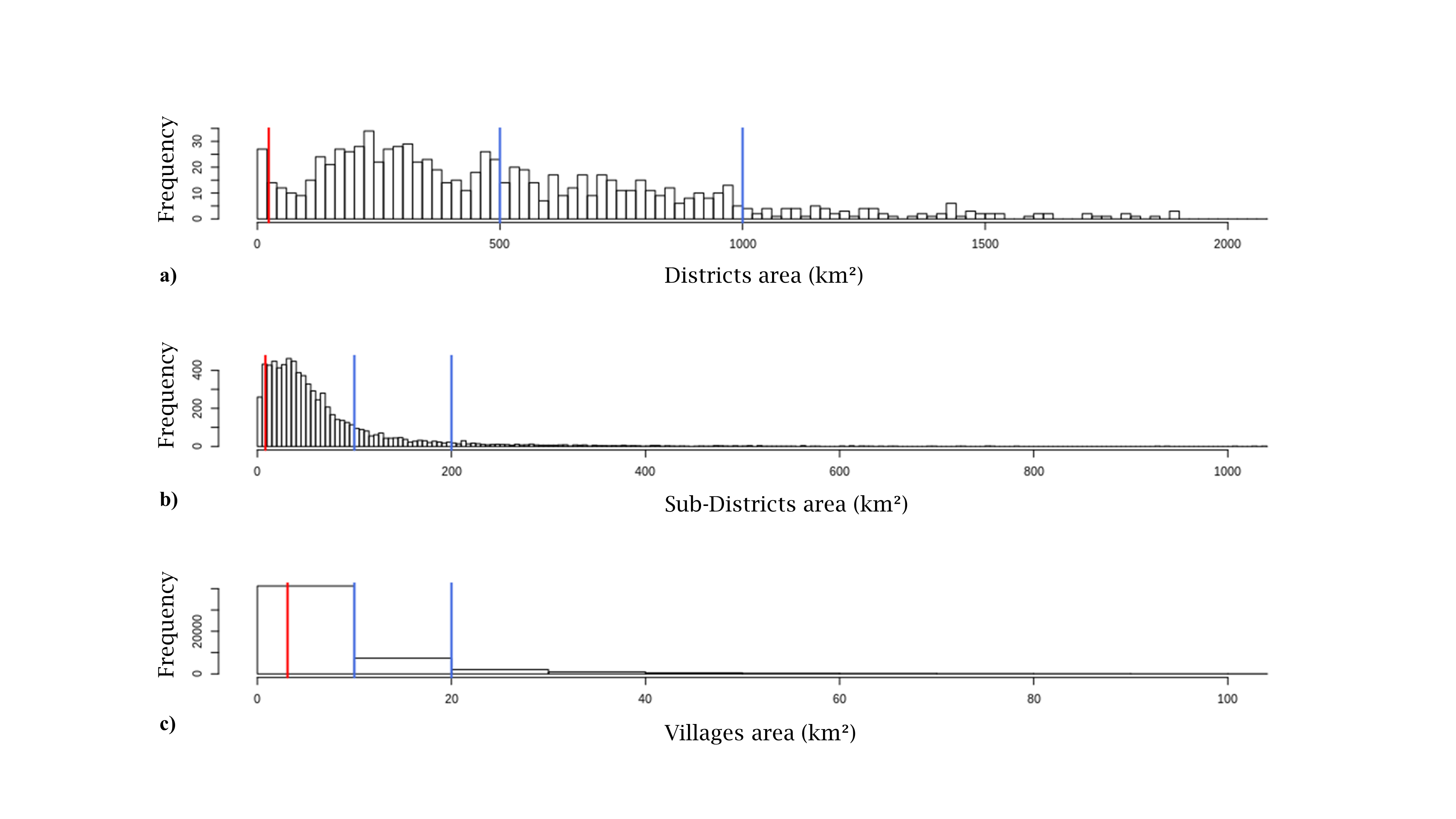

Supplement: S1 Fig — The histograms of the area of polygon sampling units used to estimate RMSE and COR for different polygon areal sizes. The red bars represent the Average Spatial Resolution (ASR) of the polygons, while the blue lines are the polygon area classes chosen: a) 0-500 km², 500-1000 km² and >1000 km² are the districts area classes used, ASR = 3.11 km, b) 0-100 km², 100-200 km² and >200 km² are the sub-districts area classes used, ASR = 8.33 km, c) 0-10 km², 10-20 km² and >20 km² are the villages area classes used, ASR = 23.60 km. (TIF) [file pone.0221070.s001.TIF]

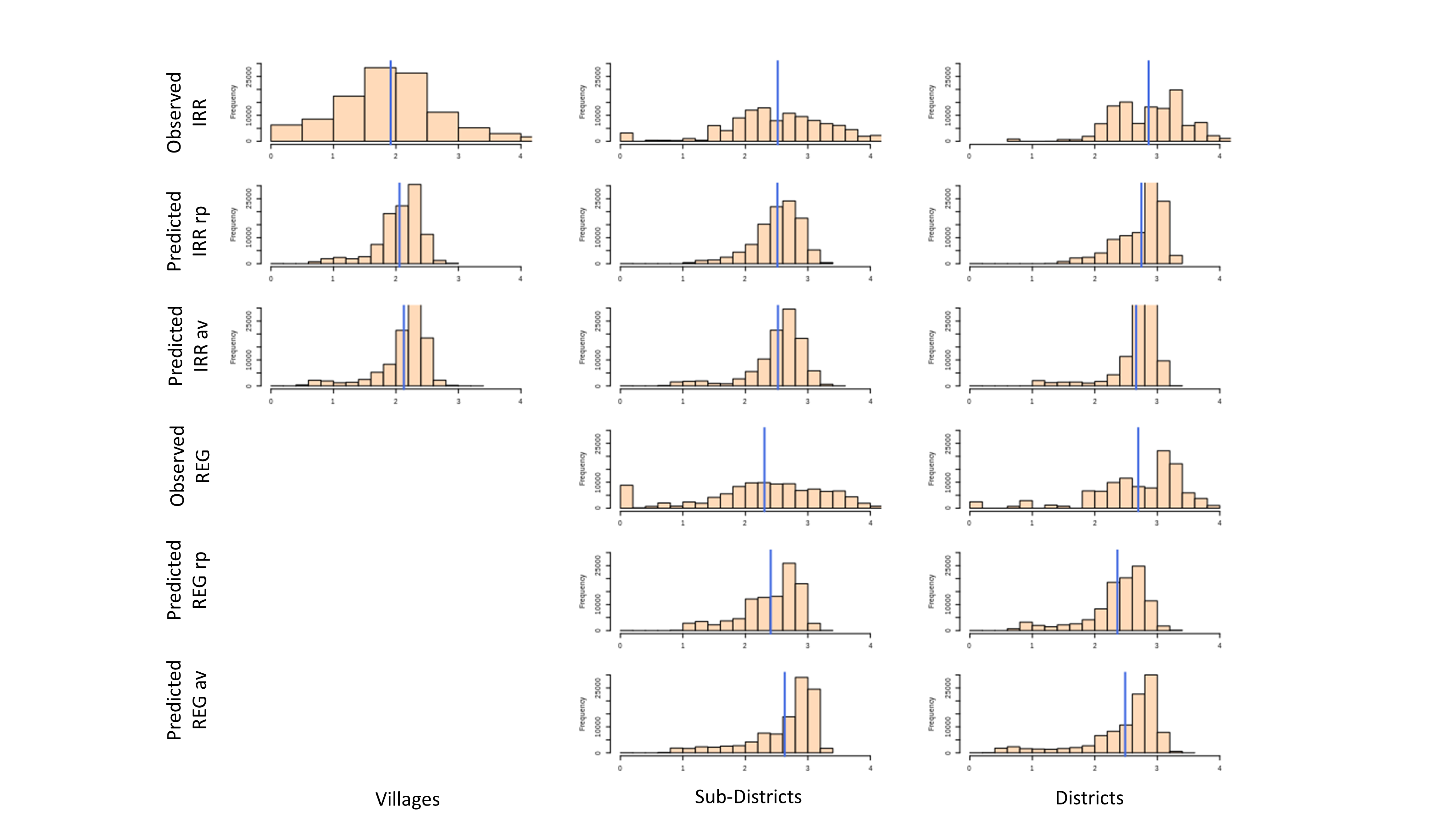

Supplement: S2 Fig — The blue lines represent the mean value. (TIF) [file pone.0221070.s002.TIF]

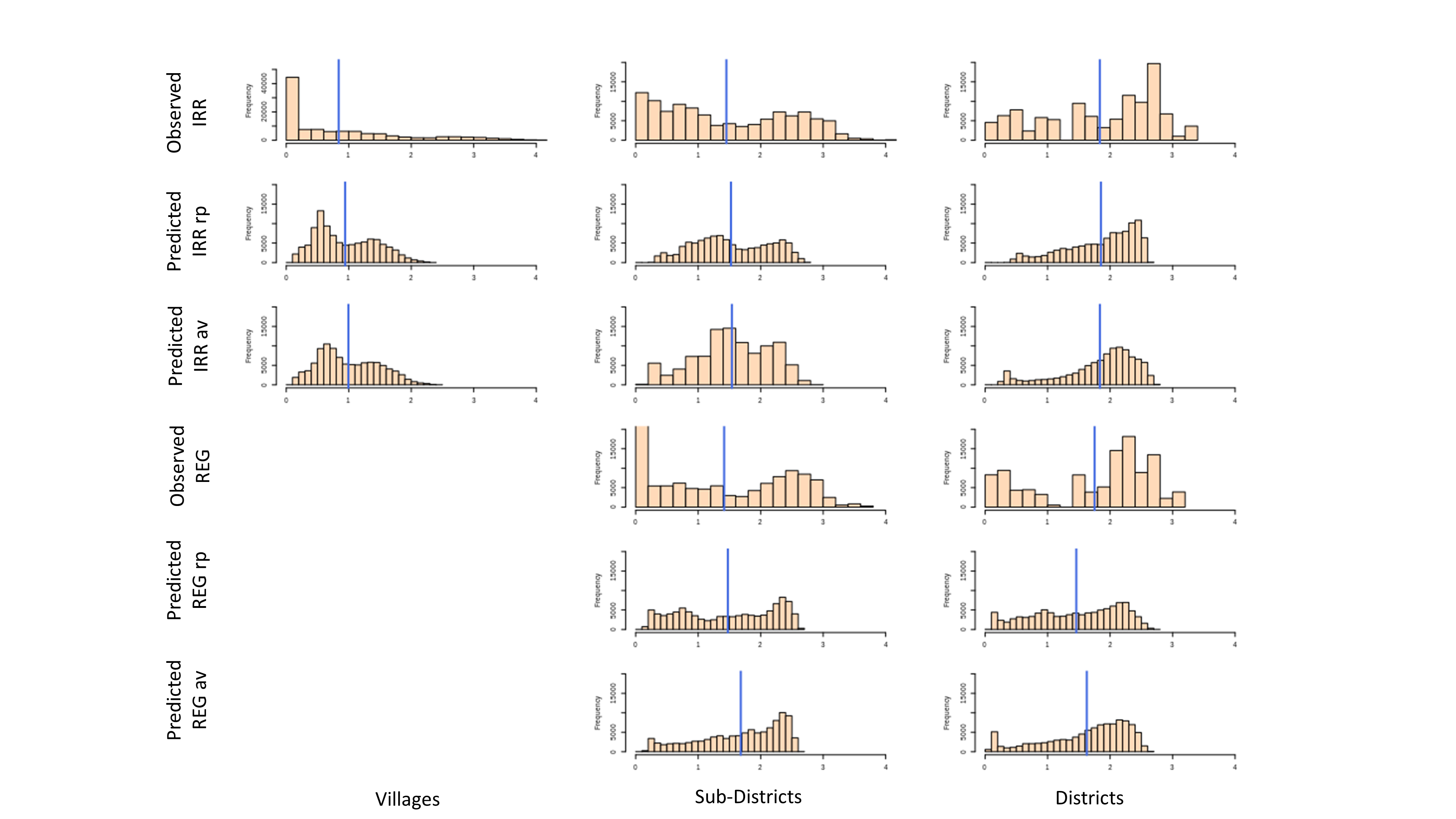

Supplement: S3 Fig — The blue lines represent the mean value. (TIF) [file pone.0221070.s003.TIF]

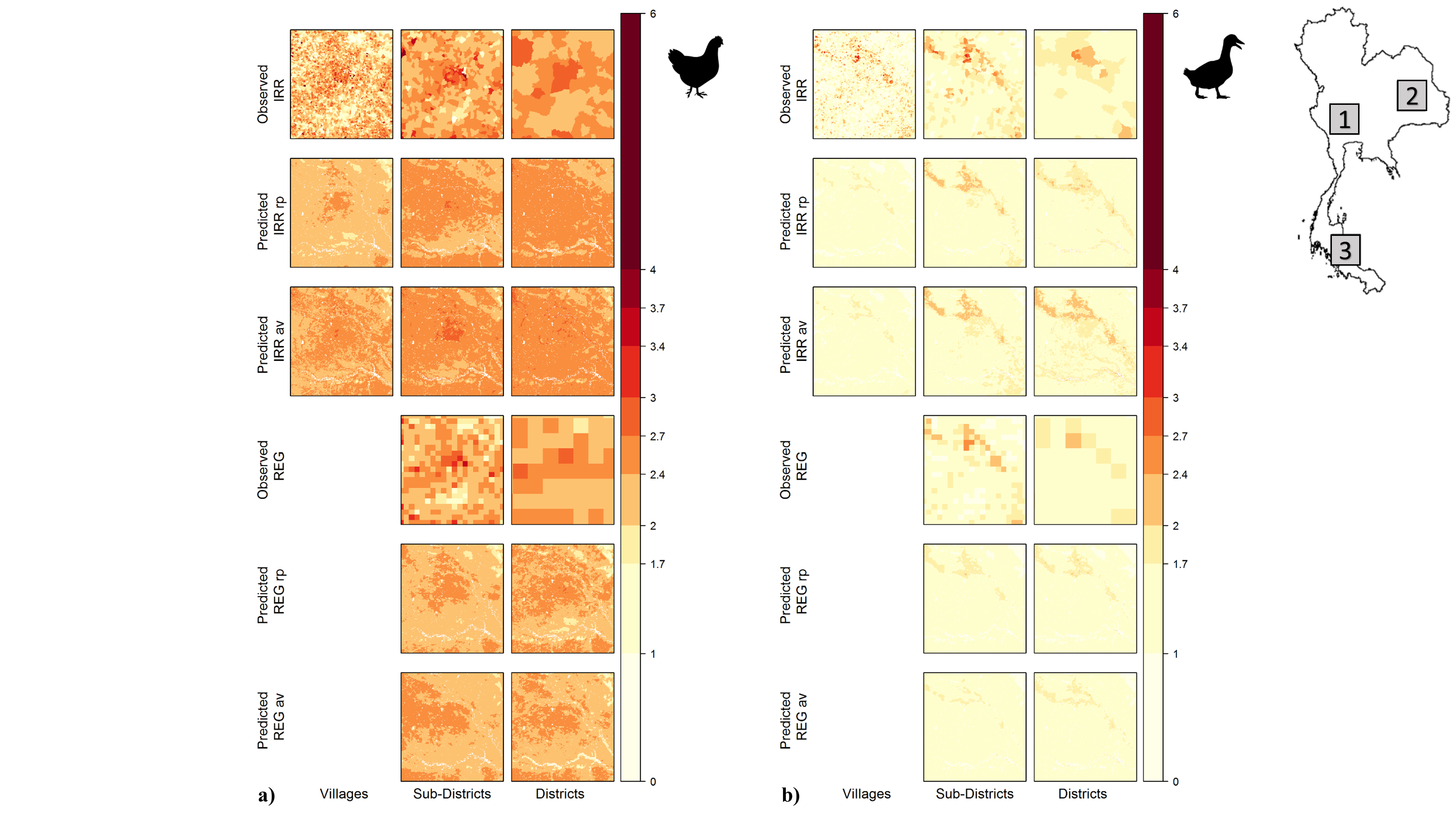

Supplement: S4 Fig — a) chickens, b) Ducks. (TIF) [file pone.0221070.s004.TIF]

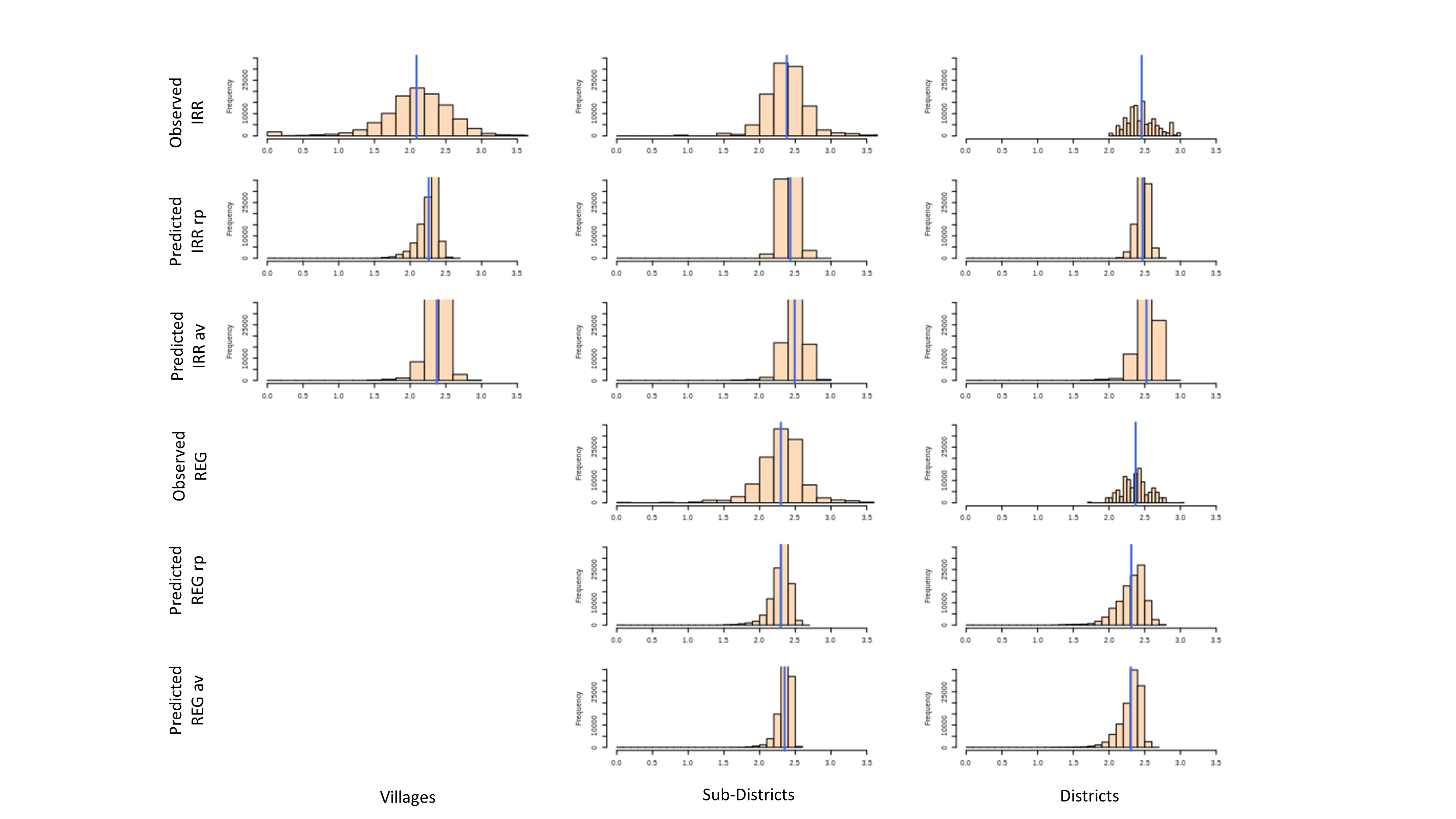

Supplement: S5 Fig — The blue lines represent the mean value. (TIF) [file pone.0221070.s005.TIF]

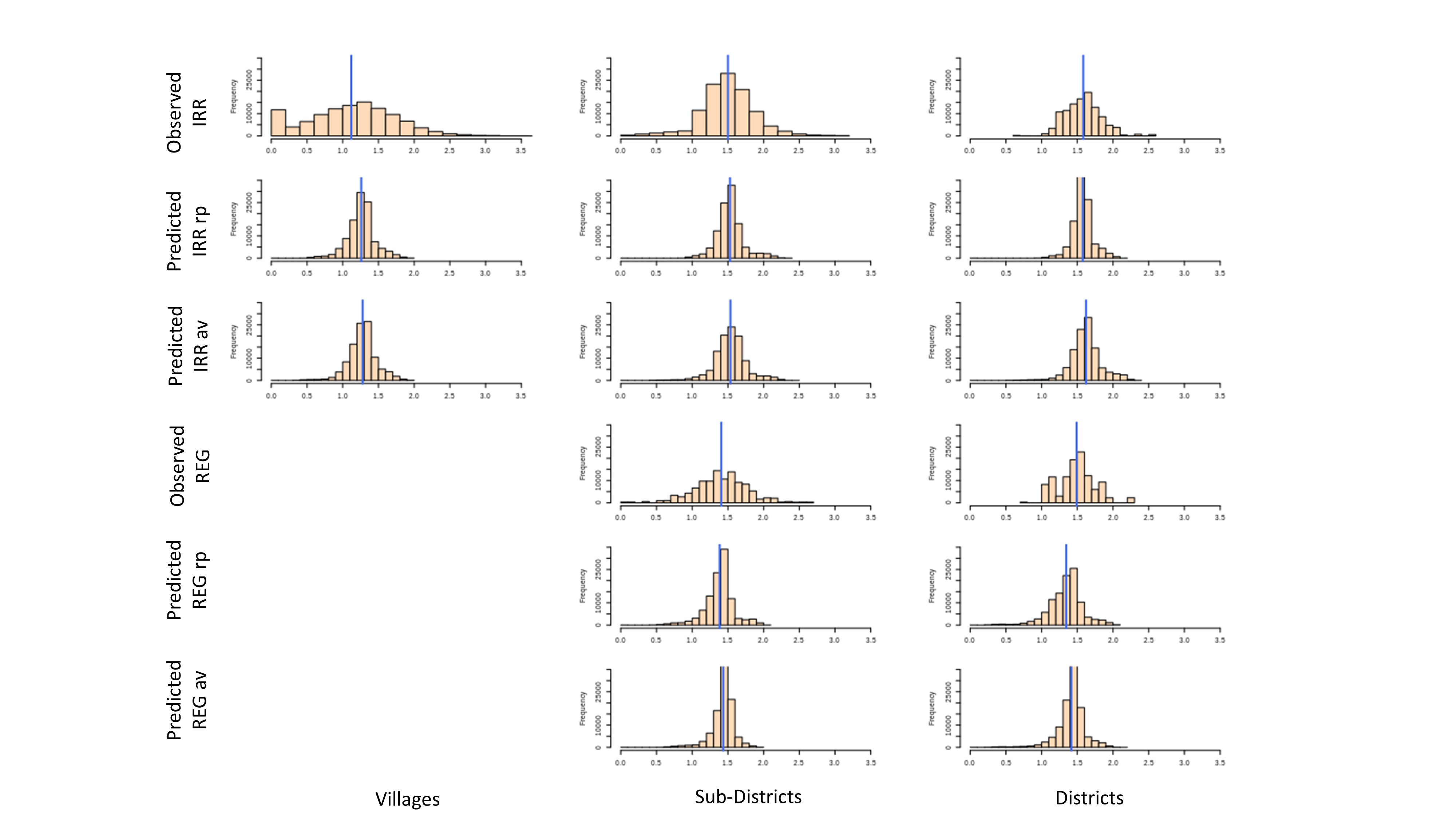

Supplement: S6 Fig — The blue lines represent the mean value. (TIF) [file pone.0221070.s006.TIF]

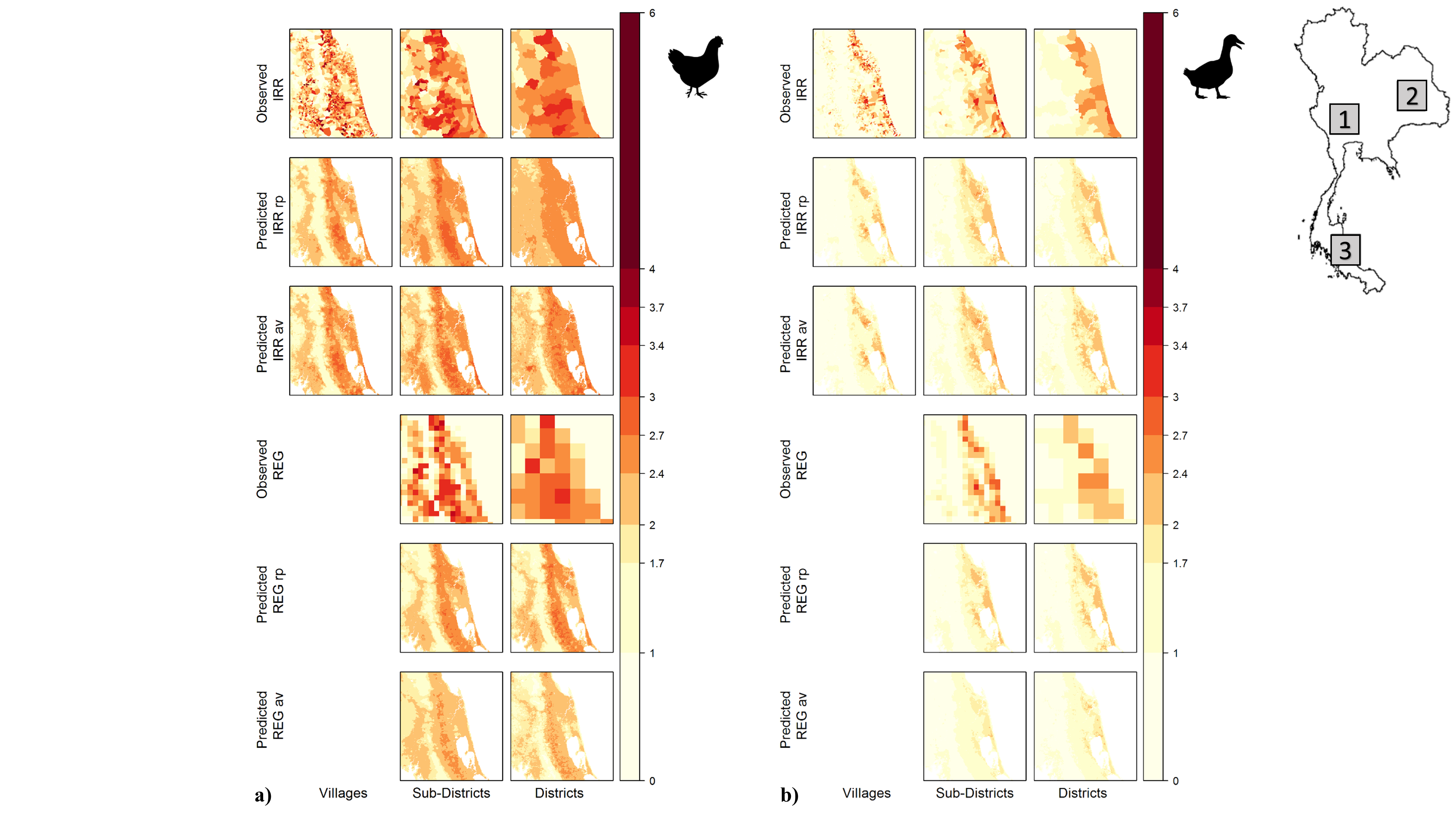

Supplement: S7 Fig — a) chickens, b) Ducks. (TIF) [file pone.0221070.s007.TIF]

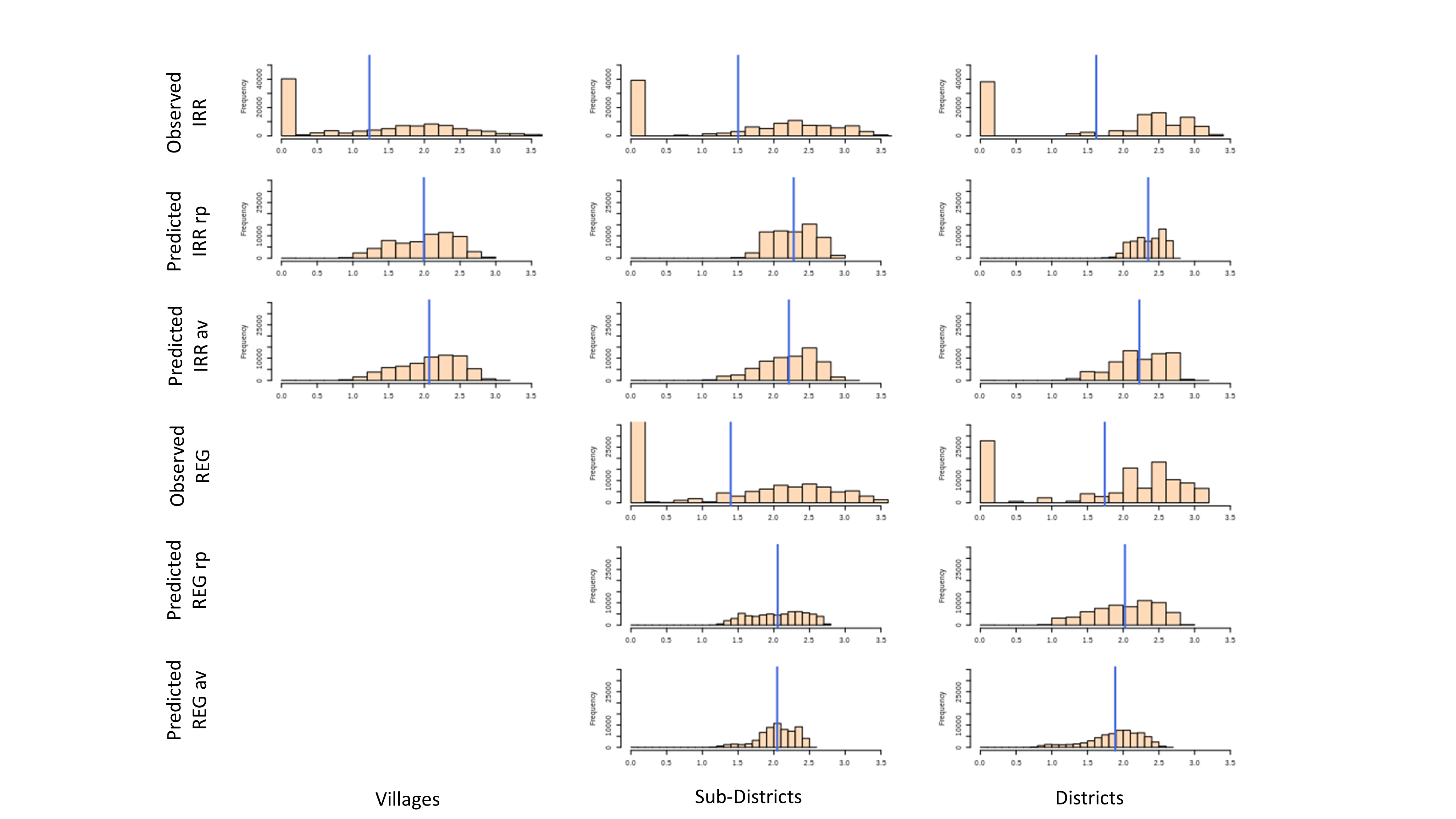

Supplement: S8 Fig — The blue lines represent the mean value. (TIF) [file pone.0221070.s008.TIF]

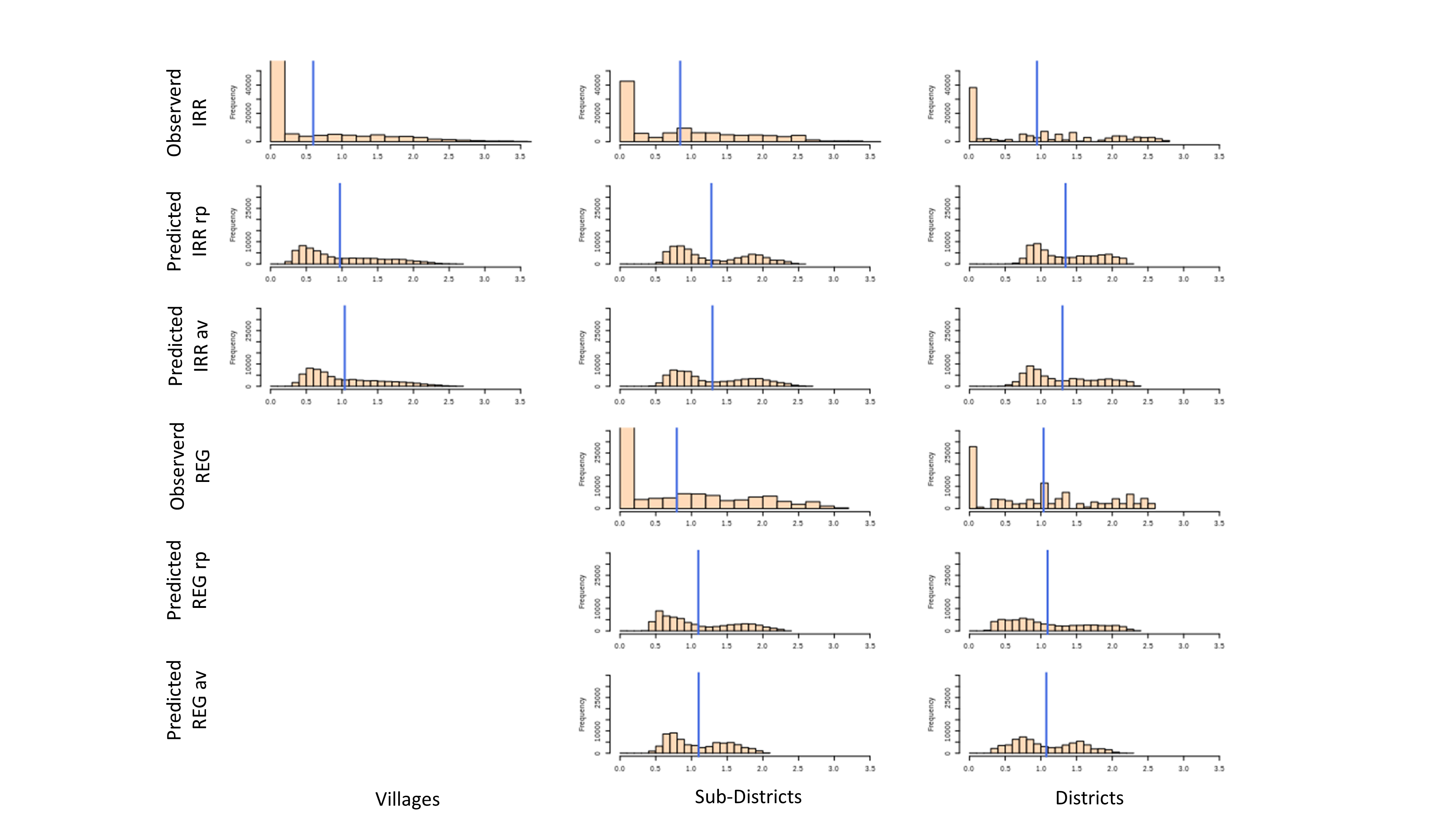

Supplement: S9 Fig — The blue lines represent the mean value. (TIF) [file pone.0221070.s009.TIF]
