# Supplementary material for: Downscaling livestock census data using multivariate predictive models: Sensitivity to modifiable areal unit problem
Source: PLoS One. 2020 Jan 27;15(1):e0221070. doi: 10.1371/journal.pone.0221070 (PMC6984718; doi:10.1371/journal.pone.0221070)
Supplement: Attachment — Submitted filename: daREgilbertetalPONE-D-19-20969_reviewed.pdf [file pone.0221070.s011.pdf]

PLOS ONE

Downscaling livestock census data using multivariate predictive models: sensitivity to modifiable areal unit problem

--Manuscript Draft--

**Manuscript Number:**	PONE-D-19-20969
**Article Type:**	Research Article
**Full Title:**	Downscaling livestock census data using multivariate predictive models: sensitivity to modifiable areal unit problem
**Short Title:**	Downscaling model sensitivity to modifiable areal unit problem
**Corresponding Author:**	Daniele Da Re<br>Université catholique de Louvain<br>Louvain-la-Neuve, Brabant-Wallon BELGIUM
**Keywords:**	MAUP; Gridded Livestock of the World; Thailand; Poultry
**Abstract:**	The analysis of census data aggregated by administrative units introduces a statistical bias known as the modifiable areal unit problem (MAUP). Previous researches have mostly assessed the effect of MAUP on upscaling models. The present study contributes to clarify the effects of MAUP on the downscaling methodologies, highlighting how a priori scales and shapes choices could influence the results. We aggregated chicken and duck fine-resolution census in Thailand, using three administrative census levels in regular and irregular shapes. We then disaggregated the data within the Gridded Livestock of the World analytical framework, sampling predictors in two different ways. A sensitivity analysis on Pearson's r correlation statistics and RMSE were carried out to understand how size and shapes of the response variables affect the goodness-of-fit and downscaling performances. We showed that scale, rather than shapes and sampling methods, affected downscaling precision, suggesting that training the model using the finest administrative level available is preferable. Moreover, datasets showing non-homogeneus distribution but instead spatial clustering seemed less affected by MAUP, yielding higher Pearson's r values and lower RMSE compared to a more spatially homogenous dataset. Implementing aggregation sensitivity analysis in spatial studies could help to interpret complex results and disseminate robust products.
**Order of Authors:**	Daniele Da Re
	Marius Gilbert
	Celia Chaiban
	Pierre BourguignonBou
	Weerapong Thanapongtharm
	Timothy P. Robinson
**Additional Information:**	
**Question**	**Response**
**Financial Disclosure**<br><br>Enter a financial disclosure statement that describes the sources of funding for the work included in this submission. Review the submission guidelines for detailed requirements. View published research articles from *PLOS ONE* for specific examples.<br><br>This statement is required for submission	D.D.R. is supported by the FRFS-WISD Walloon Institute for Sustainable Development PDR "Mapping livestock's transition" (PDR-WISD X302317F).<br><br>The funders had no role in study design, data collection and analysis, decision to publish, or preparation of the manuscript.

Powered by Editorial Manager® and ProduXion Manager® from Aries Systems Corporation

and **will appear in the published article** if the submission is accepted. Please make sure it is accurate.<br><br>**Unfunded studies**<br>Enter: *The author(s) received no specific funding for this work.*<br><br>**Funded studies**<br>Enter a statement with the following details:<br>• Initials of the authors who received each award<br>• Grant numbers awarded to each author<br>• The full name of each funder<br>• URL of each funder website<br>• Did the sponsors or funders play any role in the study design, data collection and analysis, decision to publish, or preparation of the manuscript?<br>• **NO** - Include this sentence at the end of your statement: *The funders had no role in study design, data collection and analysis, decision to publish, or preparation of the manuscript.*<br>• **YES** - Specify the role(s) played.<br><br>* typeset	
**Competing Interests**<br><br>Use the instructions below to enter a competing interest statement for this submission. On behalf of all authors, disclose any competing interests that could be perceived to bias this work—acknowledging all financial support and any other relevant financial or non-financial competing interests.<br><br>This statement **will appear in the published article** if the submission is accepted. Please make sure it is accurate. View published research articles from *PLOS ONE* for specific examples.	The authors have declared that no competing interests exist.

**NO authors have competing interests**<br><br>Enter: *The authors have declared that no competing interests exist.*<br><br>**Authors with competing interests**<br><br>Enter competing interest details beginning with this statement:<br><br>*I have read the journal's policy and the authors of this manuscript have the following competing interests: [insert competing interests here]*<br><br>* typeset	
**Ethics Statement**<br><br>Enter an ethics statement for this submission. This statement is required if the study involved:<br><br>• Human participants<br>• Human specimens or tissue<br>• Vertebrate animals or cephalopods<br>• Vertebrate embryos or tissues<br>• Field research<br><br>Write "N/A" if the submission does not require an ethics statement.<br><br>General guidance is provided below. Consult the submission guidelines for detailed instructions. **Make sure that all information entered here is included in the Methods section of the manuscript.**	N/A

Powered by Editorial Manager® and ProduXion Manager® from Aries Systems Corporation

**Format for specific study types**<br><br>**Human Subject Research (involving human participants and/or tissue)**<br>• Give the name of the institutional review board or ethics committee that approved the study<br>• Include the approval number and/or a statement indicating approval of this research<br>• Indicate the form of consent obtained (written/oral) or the reason that consent was not obtained (e.g. the data were analyzed anonymously)<br><br>**Animal Research (involving vertebrate animals, embryos or tissues)**<br>• Provide the name of the Institutional Animal Care and Use Committee (IACUC) or other relevant ethics board that reviewed the study protocol, and indicate whether they approved this research or granted a formal waiver of ethical approval<br>• Include an approval number if one was obtained<br>• If the study involved *non-human primates*, add *additional details* about animal welfare and steps taken to ameliorate suffering<br>• If anesthesia, euthanasia, or any kind of animal sacrifice is part of the study, include briefly which substances and/or methods were applied<br><br>**Field Research**<br><br>Include the following details if this study involves the collection of plant, animal, or other materials from a natural setting:<br>• Field permit number<br>• Name of the institution or relevant body that granted permission	
**Data Availability**<br><br>Authors are required to make all data underlying the findings described fully available, without restriction, and from the time of publication. PLOS allows rare exceptions to address legal and ethical concerns. See the PLOS Data Policy and FAQ for detailed information.	Yes - all data are fully available without restriction

Powered by Editorial Manager® and ProduXion Manager® from Aries Systems Corporation

A Data Availability Statement describing where the data can be found is required at submission. Your answers to this question constitute the Data Availability Statement and **will be published in the article**, if accepted.<br><br>**Important:** Stating 'data available on request from the author' is not sufficient. If your data are only available upon request, select 'No' for the first question and explain your exceptional situation in the text box.<br><br>Do the authors confirm that all data underlying the findings described in their manuscript are fully available without restriction?	
**Describe where the data may be found in full sentences. If you are copying our sample text, replace any instances of XXX with the appropriate details.**<br><br>• If the data are **held or will be held in a public repository**, include URLs, accession numbers or DOIs. If this information will only be available after acceptance, indicate this by ticking the box below. For example: *All XXX files are available from the XXX database (accession number(s) XXX, XXX.*).<br>• If the data are all contained **within the manuscript and/or Supporting Information files**, enter the following: *All relevant data are within the manuscript and its Supporting Information files.*<br>• If neither of these applies but you are able to provide **details of access elsewhere**, with or without limitations, please do so. For example:<br><br>*Data cannot be shared publicly because of [XXX]. Data are available from the XXX Institutional Data Access / Ethics Committee (contact via XXX) for researchers who meet the criteria for access to confidential data.*<br><br>*The data underlying the results presented in the study are available from (include the name of the third party*	The R codes used in the study and the aggregated census data at different administrativ level are available in the gitlab folder https://gitlab.com/danidr/glw/tree/master/glw_maup

and contact information or URL).<br>• This text is appropriate if the data are owned by a third party and authors do not have permission to share the data.<br><br>* typeset	
Additional data availability information:	

Powered by Editorial Manager® and ProduXion Manager® from Aries Systems Corporation

Manuscript
Click here to access/download;Manuscript;plos_maup.pdf

Downscaling livestock census data using multivariate predictive models: sensitivity to modifiable areal unit problem

Daniele Da Re[1,*], Marius Gilbert[2], Celia Chaiban[1,2], Pierre Bourguignon[1], Weerapong Thanapongtharm[3], Timothy P. Robinson[4,5], Sophie O. Vanwambeke[1]

1 Earth and Life Institute, Université catholique de Louvain, Louvain-la-Neuve, Belgium
2 Spatial epidemiology Lab (SpELL), Université Libre de Bruxelles, Brussels, Belgium
3 Department of Livestock Development (DLD), Bangkok, Thailand
4 Policies, Institutions and Livelihoods (PIL), International Livestock Research Institute (ILRI), Nairobi, Kenya
5 Livestock Information, Sector Analysis and Policy Branch (AGAL), Food and Agriculture Organisation of the United Nations (FAO), Rome, Italy

* daniele.dare@uclouvain.be

Abstract

The analysis of census data aggregated by administrative units introduces a statistical bias known as the modifiable areal unit problem (MAUP). Previous researches have mostly assessed the effect of MAUP on upscaling models. The present study contributes to clarify the effects of MAUP on the downscaling methodologies, highlighting how a priori scales and shapes choices could influence the results. We aggregated chicken and duck fine-resolution census in Thailand, using three administrative census levels in regular and irregular shapes. We then disaggregated the data within the Gridded Livestock of the World analytical framework, sampling predictors in two different ways. A sensitivity analysis on Pearson's r correlation statistics and RMSE were carried out to understand how size and shapes of the response variables affect the goodness-of-fit and downscaling performances. We showed that scale, rather than shapes and sampling methods, affected downscaling precision, suggesting that training the model using the finest administrative level available is preferable. Moreover, datasets showing non-homogeneus distribution but instead spatial clustering seemed less affected by MAUP, yielding higher Pearson's r values and lower RMSE compared to a more spatially homogenous dataset. Implementing aggregation sensitivity analysis in spatial studies could help to interpret complex results and disseminate robust products.

Introduction

Spatial data are becoming increasingly more accessible to the scientific community. However, much data are provided in an aggregated form at different administrative levels, mainly for operational and privacy reasons [1, 2]. Administrative levels are usually arbitrarily determined and modifiable, meaning that they can be assembled to form units of different sizes and shapes [3, 4]. Because administrative units may not adequately reflect the spatial organization of human or natural phenomena, researchers pursue the elaboration of methods for data disaggregation with the help of broadly

available remote sensing data. Often, little attention is paid to the issue of the modifiable units and its effect on spatial representations [5]. This specific issue was discussed in the spatial analysis literature since the 1930s (e.g. [6]), but gained attention with the milestone work of Openshaw and Taylor [7, 8] that led to the introduction of the concept of Modifiable Areal Unit Problem (MAUP). The MAUP encompasses two related but distinctive components: the scale issue and the zonation issue [3, 4, 7–10]. The scale problem reflects how the description of a phenomenon is potentially affected by changing the size of the sampling units, while the zonation issue relates to how changing the shape of sampling units could influence the representation of the phenomenon [7]. These effects occur because patterns and processes operate in the real world according to various scales and designs that are often unknown to the researcher [9]. A descriptive example illustrates some immediate effects. Fig 1a shows how the aggregation of individual-level data at different scales causes a reduction of the variability, and thus narrowing of the distribution. In Fig 1b, individual-level data are aggregated at the same scale but using different, arbitrary, areal unit shapes. The results are highly variable [3, 8, 10].

MAUP is closely related to the ecological inference fallacy, a misinterpretation of statistical inferences drawn at the group level but interpreted at the individuals level [11]. With spatial data becoming a staple in a diversity of fields, the effects of MAUP are explored broadle, from ecology to remote sensing and from physical geography to economy [3, 10, 12–18]. Despite the impact of MAUP is often ingnored [5], when it is addressed researchers mostly assess its effect on upscaling, or aggregating [3, 16, 18], rather than on downscaling, or disaggregating. The availability of spatial data and data processing capacity fostered an interest into the spatial heterogeneity of diverse processes and encouraged researchers to find ways to disaggregate data. Downscaling techniques are used to disaggregate variables recorded or distributed at an aggregated scale, such as census data, and provide predictions at a finer level of spatial detail. Such fine scale data bear crucial interest in diverse fields and applications in agricultural socio-economics, food security, environmental impact assessment and epidemiology [19]. Concerning livestock, analyzing the emergence of zoonotic diseases requires detailed spatially explicit data of both hosts and their pathogens, e.g. for pathogenic avian influenza (HPAI, [20]).

The Gridded Livestock of the World (GLW, [21]) and WorldPop [22] disaggregate population data using statistical techniques and environmental predictors. Outputs of both projects reach good accuracy scores [19, 23], but as they result from a downscaling process, both are potentially subjected to the MAUP. Despite the application of the GLW methodology has become robust and its application frequent (e.g. [19, 24, 25], its dependencies to MAUP has not been directly investigated yet. Previous studies (e.g. [24]) showed a certain degree of sensitivity to the scale issue, however, the severity of the problem has not been assessed and a sensitivity analysis using various scale and shape configurations would help quantifying potential sources of uncertainties.

In this study, we analyzed the impact of both MAUP effects on the disaggregation of census-like livestock data. The objectives were: (i) to assess, on two different spatially-constrained real datasets, how the MAUP affects both goodness-of-fit metrics and downscaled results, (ii) to increase awareness about the MAUP issues in the context of data disaggregation. A fine resolution census dataset of poultry in Thailand was aggregated at scales corresponding to administrative levels, using sampling units with variable shapes and areas and subsequentely disaggregated to a common resolution over a 500m grid.

Fig 1. The Modifiable Areal Unit Problem. Example showing the two effects of MAUP (adapted from [3]).

Materials and methods

Poultry population data

In 2010, the Department of Livestock Development of the Thai government conducted a national census of poultry in each sub-district and village, counting poultry heads per owner. Each farm was associated by a unique administrative code number to its village, for which geographic coordinates was recorded. The census distinguished between broiler chickens, layer chickens, native chickens, farm ducks and free-grazing ducks. Here, we combined all data to species level ending with chicken and duck. The spatial constraints and determinants of the production systems of duck and chickens differ (intensive and backyard; [26–28]). While chickens can be raised anywhere, in Thailand, ducks are largely raised in wetlands used for double-crop rice production, where free-grazing ducks feed year round in rice paddies [27, 28].

Villages records with uncorrected coordinates (coordinates outside of the Thai territory or with 0 in latitude or longitude fields) were removed. In the case of duplicate coordinates or duplicate village unique ID, only one record for each duplicate was randomly selected. The provinces of Bangkok, Nakhon Sawan, Pattani and Phetchaburi were excluded due to lack of data. Once filtered, the village dataset was joined to the census datas using the villages' administrative code number.

Individual level data were aggregated according to Thai administrative units: districts, sub-districts and villages. As a comprehensive file of village boundaries is not available, V[illegible]onoi polygons were computed from the village coordinates.

Modelling

We used the methodology of the Gridded Livestock of the World (GLW) project. The GLW disaggregates livestock statistics and provides spatially detailed estimates of livestock density in the form of raster spatial data [21]. The most recent version (GLW3; [25]) relies on stratified random forest models and a set of environmental predictors. The GLW methodology is fully described in [24] and [19]. Two user-controlled parameters drive the performance of random forest models: the number of trees created and the number of variables randomly selected when creating a splitting point. [29] have shown that 500 trees are a good rule of thumb, while the minimum number of variables that are randomly selected was calculated using the square root of the total number of variables [30].

The set of predictors used included Fourier-transformed MODIS variables (two vegetation indices, the day and night land surface temperature and the band 3 middle-infra-red), eco-climatic variables (length of growing season and annual precipitation), topographic variables (elevation and slope) land cover classes and anthropogenic variables (human population density and travel time to major cities and ports) [19, 27, 28, 31].Unpopulated areas, natural areas and water bodies were masked out and only areas suitable for poultry production were considered and used to get corrected poultry densities. Poultry densities corrected by area were transformed to logarithm (base 10) and used as response variable. The full list of spatial domain and predictors is detailed in Table 1 along with sources.

All input raster layers (e.g. masks and predictor variables) and outputs (predicted densities) were processed on the whole of Thailand with a spatial resolution of 500 m.

Experimental design

The effect of scale was explored by aggregating the individual level data to village, sub-district and district level. The effects of zoning were analyzed using two different

Table 1. List of input spatial dataset used to model chickens and ducks densities.

Type	Variables	Use	Source
Land	Land and water area	Spatial domain and Spatial predictor	[32,33]
Land use	IUCN world database of protected area	Mask	[34]
Anthropogenic	Worldpop Human population density	Spatial predictor and suitability mask	[22]
	Travel time to cities capital, Provinces' capital and main harbors	Spatial predictor	[35,36]
Topography	Elevation (GTOPO30)	Spatial predictor	[37]
	Slope (GTOPO30)	Spatial predictor	[37]
Vegetation	10 Fourier-derived variables from Normalized Difference Vegetation Index from MODIS (MODIS)*	Spatial predictor	[38]
	Length of growing period	Spatial predictor	[39]
	Green-up and senescence (annual cycle 1 and 2)	Spatial predictor	[40]
	Forest cover	Spatial predictor	[41]
	Cropland, irrigated croplands and rainfed croplands cover	Spatial predictor	[42]
Climatic	10 Fourier-derived variables from Day/Night Land Surface Temperature (MODIS)	Spatial predictor	[38]
	Precipitations	Spatial predictor	[43]

*Annual mean, annual muinimum, annual maximum, amplitude and phase of annual cycle, amplitude and phase of bi-annual cycle, amplitude and phase of tri-annual cycle, variance in annual, bi-annual, and tri-annual cycles..

sets of polygon sampling units (PSUs) for each administrative level: (i) irregular (IRR) shapes, the original administrative units, and (ii) regular shapes (REG), a grid having the spatial resolution of the average spatial resolution (ASR) of the correspondent IRR PSUs. The ASR measures the effective resolution of administrative units in kilometers. It is calculated as the square root of the land area of the administrative units considered, divided by the number of administrative units [44,45]. REG PSUs were computed only at sub-district and district level. The density of birds per km^2 of suitable land was estimated in all polygons corresponding to each PSUs and transformed to its Log10 [24].

Two methods where applied to extract or sample the predictors by polygon, in order to understand their effect on the downscaled prediction. One method randomly sampled a point in each PSU and extracted the matching pixel value for each predictor. The other averaged the predictors within the PSU.

Model evaluation

The census polygons used as response variable were separated in training and validation sets. 70% of polygons were used to train the model, while the remaining 30% were used as evaluation data set. PSUs were sampled into training and evaluation datasets 20 times to assess the internal variability of the predictions. Once the model was fitted, average and standard deviation maps were computed from the twenty outputs.

Model evaluation was carried out using two approaches. Firstly, to assess how well the model predicted poultry densities, the root mean square error (RMSE) and Pearson's r correlation coefficient (COR) were computed between the observed values of the evaluation set of PSU and the predicted densities aggregated at polygon level of the corresponding validation PSUs. RMSE measures model accuracy, i.e. how far the predicted values were, on average, from the observed values. COR measures precision, i.e. the extent to which the observed and predicted values are proportional to each other. Lower RMSE and higher COR indicate better fits between predicted and observed values. RMSE and COR were estimated for the overall models. Moreover, to measure the internal precision associated to the area, RMSE and COR were also estimated considering PSUs area, grouping PSUs accordingly to the frequencies of their area (Supporting information, S1 Fig): 0-10 km^2, 10-20 km^2 and >20 km^2 for villages, 0-100 km^2, 100-200 km^2 and >200 km^2 for sub-districts, 0-500 km^2, 500-1000 km^2 and >1000 km^2 for districts.

Secondly, the Pearson's r was computed between predictions and the observed data at the village level only to assess the capacity of models trained using various PSUs to predict poultry population at a fine scale, i.e their "downscaling precision" (COR_{down}).

This is crucial to understand the effects of MAUP on the downscaled predictions considering the finest administrative levels available as reference. Three different bounding boxes (hereafter *bbox*) were selected in different areas of Thailand to visually investigate the differences between the predictions and the observations. A graphical summary of the methodology is shown in Fig 2. The model is fully operational under R 3.4 [46] and the codes used, as well as the aggregated census data, are available in S1 Code.

Fig 2. Flowchart of the analysis.

Results

Data cleaning and filtering

The 62 142 village records originally available were reduced to 57 794 (Table 2). Once the filtered village database was joined to the poultry census, the final georeferenced census dataset used to train the models accounts for 53 301 records (Table 2). Fig 3 show the two species observed densities aggregated at sub-districts and districts administrative level. Chickens were homogenously distributed. Ducks were mainly clustered in the central and southeast part of the country.

Table 2. Data filtering results. For duplicate coordinates or duplicate village unique ID, only one record for each duplicated row was randomly selected and added to the finale database.

	Unfiltered	Duplicated ID	Duplicated coordinates	Filtered
Villages	62142	6579	33	57794
Census	3170213	-	-	53301

Fig 3. Observed poultry densities in logarithm (base 10) aggregated at Districts and Sub-Districts level. In grey the provinces of Bangkok, Nakhon Sawan, Pattani and Phetchaburi, excluded from the analysis due to lack of data.

Model outputs maps

The model predictions within *bbox* 1 are shown in Fig 4, while *bbox* 2-3 are displayed in the S3 Fig and S4 Fig. Chickens were widely distributed though high densities clusters are observable in the North-East and South-West parts of *bbox* 1. Ducks were present mostly in the central part. The model was able to reproduce the observed spatial pattern of both species, regardless of the sampling method.

The mean predicted values are comparable to the observed ones but the predicted values distributions are clustered around the mean and appeared less variable than the observed. For both species, the aggregation of input data produced higher mean value at coarser scale, together with a narrowing effect of the values distribution and a smoothing effect on the frequencies (S2.1 Fig and S2.2 Fig).

IRR and REG shaped administrative units showed slightly different predicted spatial pattern. In both cases, the distribution of the predicted values is consistent with the observed values, however, REG shapes seemed to predict a slightly smoother spatial pattern, detecting more variability across space than IRR shapes, which predicted more values clustered around and above the mean value.

Fig 4. Observed and Predicted Log10 poultry values inside *bbox*1. a) chickens, b) Duck.

Model evaluation

The RMSE bar plots for ducks and chickens are shown in Fig 5. For both species, the overall accuracy increased (lower RMSE values) as the administrative level of the input data became coarser. However, this trend is more consistent for ducks rather than for chickens. Model runs on REG shaped PSUs showed generally less variability, but they had lower accuracy than IRR PSUs for chickens and comparable or slightly lower for ducks. Randomly sampling the predictors within the PSUs yielded slightly lower RMSEs than their aggregation.

COR bar plots based on stratified random sampling of the predictors and averaged predictors are a shown in Fig 6. For both species, the COR value increased as the administrative level of the input data became coarser. REG PSUs produced higher correlations than the corresponding IRR PSUs and the overall models, showing also less variability among the bootstraps. The choice of the sampling methods did not affect the results strongly, but random sampling showed apparently higher variability between individual bootstraps.

Fig 5. Root Mean Square Error (RMSE). RMSE computed between predicted densities and observed chickens densities a) averaged sampling b) random sampling; RMSE computed between predicted densities and observed ducks densities c) averaged sampling d) random sampling.

Fig 6. Pearson's *r*. Pearson's r coefficient computed between predicted densities and observed chickens densities a) averaged sampling b) random sampling; Pearson's r coefficient computed between predicted densities and observed ducks densities c) averaged sampling d) random sampling.

Downscaling precision

COR_{down}, the Pearson's r coefficient between the predicted and observed densities at village level are shown in Fig 7. Models of duck distribution had higher correlations than the chickens models. Contrary to the internal precision of the model, smaller PSUs had higher Pearson's r values than larger ones. The shape of the PSUs produced comparable results in terms of Pearson's r values. Random sampling produced higher Pearson's r values compared to average sampling, which generally had a lower variability among model runs. A table summarising the evaluation of model runs is found in S1 Table.

Fig 7. Downscaling precision. Pearson's r coefficient between predicted densities and observed densities at village level: a) chickens; b) ducks. Random sampling (rp), averaged sampling (av).

Discussion

Overall MAUP bias

Our model predicted poultry density patterns and value distribution similar to the observed densities, confirming the validity of the methodology [19]. As expected,

chickens were dispersed at high densities across the whole country, while ducks were constrained to wetlands used for double-crop rice production [20, 27, 28].

The scale of the training data affected the output maps goodness-of-fit. On average, duck models showed higher downscaling precision and higher accuracy and precision compared to chickens. Swift, Liu and Uber [47] and Swift et al. [14] reported that a spatially clustered phenomenon aggregated using various size and shapes of areal units is less affected by MAUP compared to a randomly distributed phenomenon. Because of that, when the clustered structure of the observed point pattern is preserved, MAUP bias is considerably reduced. Moreover, Swift et al. [14] also showed that aggregating the independent variable using an areal unit shape related to its spatial structure reduce the effect of MAUP, in their case it worked for simulated data only. To aggregate empirical data, choosing a priori areal unit shape that preserves the spatial structure and reduces the MAUP may be challenging, and in the context of data disaggregation, may be impossible. But, in the context of data disaggregation, the MAUP bias may be smaller if the spatial units are able to capture the spatial variability of the phenomenon at hand. Recently Tuson et al. [48] proposed a theorethical and statistical framework to address the MAUP trying to detect a minimal geographical unit of analysis. Though promising, in our case the minimal geographical unit of analysis is determined by the minimal administrative level available, making the results dependent on the units used.

MAUP scale effect

Qualitatively, fine resolution training data produced predictions with a more detailed spatial pattern compared to coarser resolution tr[illegible]ng data. For what concerns the effect of scale on the internal precision of the mo[illegible] better model precision and accuracy was reached by models trained with coarser resolution input data, contrary to what Van Boeckel et al. [20] found. These apparently contradictory results can be explained considering that Van Boeckel et al. [20] used different modelling approaches and that their goodness-of-fit were computed under a different rationale. In particular, whilst our goodness-of-fit metrics were computed between validation PSUs and predicted pixel values aggregated at the respective PSUs areas, Van Boeckel et al. [20] computed goodness-of-fit metrics between validation and predicted value at point level. Though the RMSE and COR trends are not in accordance with this previous study on Thai poultry, our results are consistent with their findings in terms of RMSE and COR ranges. More importantly, our results reflect the general trend described by Gehlke and Biehl [6], where correlation coefficients tend to increase as the number of areal units representing the data decreased, as a consequence of the data smoothing properties associated with the aggregation process.

MAUP zone effect

Comparing COR and RMSE results at the same scale, REG PSU produced slightly higher mean values and less variability between model runs than IRR ones (S1 Table). In our case Pearson's r is the statistic most affected by the zone effect, but still it appeared marginal in comparison to the effect of scale, as observed also by Swift et al. [14] for simulated data. Recently, [18] investigated the effects of MAUP using landscape heterogeneity as a proxy of species richness. They highlighted how the use of irregularly shaped eco-geographic area units (watersheds) performed better than arbitrary square units, probably because in their case eco-geographic areas better capture species diversity spatial variability. Though our REG PSU based on the ASR of the IRR PGU showed higher precision scores, our design remains affected by ecological fallacy, as both administrative levels and PSU shapes may be indepedentend of the phenomena investigated and not effectively describe the environmental and social

envelope of farm distribution in geographical space [11, 49]. On this point, Fox et al. [49] suggest that combining reasonable assumptions to empirical data and spatial analysis may help to develop functional boundaries around the individual level investigated.

Sampling methods effect

The choice of the sampling methods of the predictors did not affect RMSE and both correlation coefficients. The mean value of our evaluation indices were stable and the variability observable in Fig 5,6,7 is likely more related to variability between model runs rather than to the choice of the sampling methods.

Downscaling precision

The downscaling precision statistic was affected mainly by the scale rather than by the zone or sampling methods. The ranges are generally narrow, considering scale, zone and sampling effect. The downscaling precision as expressed by COR_{down} increase with higher resolution of the training data. In fact, Robinson et al. [24] computed the goodness-of-fit metrics of their downscaling models comparing the predicted values to the observed data at the highest administrative level (a similar approach to what we did here for the COR_{down}), using a real census livestock dataset as we did. Accordingly to our findings, they underlined how the statistical model trained on smaller administrative units got better accuracy and precision in the disaggregation of administrative units. These findings suggest that, if possible, data should be collected at the finest spatial resolution available to train the model.

The question of how to select the spatial scale of the prediction according to the available detail of aggregated data remains. The choice of the spatial scale of analysis influences the understanding of the geographical patterns [50]. When downscaling it is thus crucial to understand whether the polygons' area within each administrative level could influence the disaggregated results. For instance, considering the frequency histogram of the District's areas (S1 Fig), we do not know how larger polygons affect the downscaling precision. From one perspective, adding larger polygons would include more environmental heterogeneity in the model and would allow the model to discriminate better between suitable and unsuitable area. However, since smaller polygons suit best in terms of downscaling precision, larger polygons could add noise to the spatial distribution of the response variable. It is unlikely that geometry of one set of areal units would match any measured phenomena exactly as it is and as it would occur for a simulated pattern [14], but new approaches combining geostatistics and Bayesian hierarchical models (e.g. [51–53]) are promising tools to address the MAUP effects.

Conclusion

Within the GLW framework, we assessed the MAUP effects on the downscaled predictions starting from different aggregated response variable scales. We focused on the predictive rather than the explanatory power of the model, unlike numerous studies on MAUP focused on its effects on parameter estimates or p-values (e.g. [54–57]). The goal of the downscaling methodologies is not only to compare and interpret the pixel-wise absolute value per se, but also to detect and represent well the spatial variation and pattern of the phenomena investigated. Since absolute values and trends are different, the choice of COR_{down} was made under the rationale to look for the scale that best preserves the observed value, allowing at same time to detect the existent spatial trends.

GLW is an efficient approach to disaggregate census data to predict spatial distribution of livestock. Scale, rather than shapes and sampling methods, appears to affect downscaling precision, suggesting that the finest administrative level should be sought to train the model. Moreover, MAUP effects appear weaker on a spatially constrained dataset rather than a more spatially homogenous one, as already shown for simulated data.

Carrying a sensitivity analysis and reporting the various results obtained from different sets of aggregation and zoning systems helped to adequately addressed the MAUP issue and to understand how much it affected the predictions. Understanding the magnitude of the bias introduced in the data due to the aggregation is crucial to inform spatial scientist on the often-ignored effect of data aggregation and to provide robust spatial prediction to policy maker. MAUP effect on aggregated data is unavoidable and only individual level data can avoid it [14, 58].

As already stated by previous authors(e.g. [14, 47, 59], sensitivity to aggregation should be analysed in any spatial study in order to correctly interpret complex results and disseminate clear and robust maps.

Supporting information

S1 Code GitLab repository:
`https://gitlab.com/danidr/glw/tree/master/glw_maup`

S1 Fig. Polygon sampling units areas' histograms. Polygon sampling units areas' histograms used to estimate RMSE and COR different polygon areal sizes. The red bars represent the Average Spatial Resolution (ASR) of the polygons, while the blue lines are the polygon area classes chosen: a) 0-500 km^2, 500-1000 km^2 and >1000 km^2 are the Districts area classes used, ASR = 3.11 km, b) 0-100 km^2, 100-200 km^2 and >200 km^2 are the Sub-Districts area classes used, ASR = 8.33 km, c) 0-10 km^2, 10-20 km^2 and >20 km^2 are the Villages area classes used, ASR = 23.60 km.

S2.1 Fig. Observed and Predicted Log10 chickens values histogram inside *bbox*1. The blue lines represent the mean value.

S2.2 Fig. Observed and Predicted Log10 ducks values histogram inside *bbox* 1. The blue lines represent the mean value.

S3 Fig. Observed and Predicted Log10 poultry values inside *bbox* 2. a) chickens, b) Ducks.

S3.1 Fig. Observed and Predicted Log10 chickens values histogram inside *bbox* 2. The blue lines represent the mean value.

S3.2 Fig. Observed and Predicted Log10 ducks values histogram inside *bbox* 2. The blue lines represent the mean value.

S4 Fig. Observed and Predicted Log10 poultry values inside *bbox* 3. a) chickens, b) Ducks.

S4.1 Fig. Observed and Predicted Log10 chickens values histogram inside *bbox* 3. The blue lines represent the mean value.

S4.2 Fig. Observed and Predicted Log10 ducks values histogram inside *bbox* 3. The blue lines represent the mean value.

S1 Table. Summary table of models' goodness of fit and downscaling precision.

Acknowledgments

We thank the staff of Thailand's Department of Livestock Development (DLD), composed of the District Livestock Offices, Provincial Livestock Offices, and Center for Information Technology for animal census data; Thailand's Ministry of Transportation for geodata; and theDepartment of Provincial Administration, Ministry of Interior, for population data.

Computational resources have been provided by the supercomputing facilities of the Université catholique de Louvain (CISM/UCL) and the Consortium des Equipements de Calcul Intensif en Fédération Wallonie Bruxelles (CECI) funded by the Fond de la Recherche Scientifique de Belgique (FRS-FNRS).

References

1. Goodchild MF, Proctor JD. Goodchild and Proctor 1997 Scale.pdf. Geographical and Environmental Modelling. 1997;1(1):5–23.

2. Sleeter R, Gould MD. Geographic information system software to remodel population data using dasymetric mapping methods; 2007.

3. Jelinski DE, Wu J. The modifiable areal unit problem and implications for landscape ecology. Landscape Ecology. 1996;11(3):129–140. doi:10.1007/BF02447512.

4. Marceau DJ. The Scale Issue in the Social and Natural Sciences. Canadian Journal of Remote Sensing. 1999;25(4):347–356. doi:10.1080/07038992.1999.10874734.

5. Manley D. Scale, aggregation, and the modifiable areal unit problem. Handbook of regional science. 2014; p. 1157–1171.

6. Gehlke CE, Biehl K. Certain Effects of Grouping upon the Size of the Correlation Coefficient in Census Tract Material. Journal of the American Statistical Association. 1934;29(185A):169–170. doi:10.1080/01621459.1934.10506247.

7. Openshow S. A million or so correlation coefficients, three experiments on the modifiable areal unit problem. Statistical applications in the spatial science. 1979; p. 127–144.

8. Openshaw S. Ecological Fallacies and the Analysis of Areal Census Data. Environment and Planning A: Economy and Space. 1984;16(1):17–31. doi:10.1068/a160017.

9. Manley D, Flowerdew R, Steel D. Scales, levels and processes: Studying spatial patterns of British census variables. Computers, Environment and Urban Systems. 2006;30(2):143–160. doi:10.1016/J.COMPENVURBSYS.2005.08.005.

10. Dark SJ, Bram D. The modifiable areal unit problem (MAUP) in physical geography. Progress in Physical Geography: Earth and Environment. 2007;31(5):471–479. doi:10.1177/0309133307083294.

11. Robinson W. Ecological Correlations and the Behavior of Individuals. American Sociological Review. 1950;15(3).

12. Briant A, Combes PP, Lafourcade M. Dots to boxes: Do the size and shape of spatial units jeopardize economic geography estimations? Journal of Urban Economics. 2010;67(3):287–302. doi:10.1016/J.JUE.2009.09.014.

13. Amici V, Rocchini D, Filibeck G, Bacaro G, Santi E, Geri F, et al. Landscape structure effects on forest plant diversity at local scale: Exploring the role of spatial extent. Ecological Complexity. 2015;21:44–52. doi:10.1016/J.ECOCOM.2014.12.004.

14. Swift A, Liu L, Uber J. MAUP sensitivity analysis of ecological bias in health studies. GeoJournal. 2014;79(2):137–153. doi:10.1007/s10708-013-9504-z.

15. Bacaro G, Rocchini D, Diekmann M, Gasparini P, Gioria M, Maccherini S, et al. Shape matters in sampling plant diversity: Evidence from the field. Ecological Complexity. 2015;24:37–45. doi:10.1016/j.ecocom.2015.09.003.

16. Nouri H, Anderson S, Sutton P, Beecham S, Nagler P, Jarchow CJ, et al. NDVI, scale invariance and the modifiable areal unit problem: An assessment of vegetation in the Adelaide Parklands. Science of the Total Environment. 2017;584-585:11–18. doi:10.1016/j.scitotenv.2017.01.130.

17. Salas-Olmedo MH, Moya-Gómez B, García-Palomares JC, Gutiérrez J. Tourists' digital footprint in cities: Comparing Big Data sources. Tourism Management. 2018;66:13–25. doi:10.1016/j.tourman.2017.11.001.

18. García-Llamas P, Calvo L, De la Cruz M, Suárez-Seoane S. Landscape heterogeneity as a surrogate of biodiversity in mountain systems: What is the most appropriate spatial analytical unit? Ecological Indicators. 2018;85(November 2017):285–294. doi:10.1016/j.ecolind.2017.10.026.

19. Nicolas G, Robinson TP, Wint GRW, Conchedda G, Cinardi G, Gilbert M. Using Random Forest to improve the downscaling of global livestock census data. PLoS ONE. 2016;11(3):1–16. doi:10.1371/journal.pone.0150424.

20. Van Boeckel TP, Prosser D, Franceschini G, Biradar C, Wint W, Robinson T, et al. Modelling the distribution of domestic ducks in Monsoon Asia. Agriculture, Ecosystems and Environment. 2011;141(3-4):373–380. doi:10.1016/j.agee.2011.04.013.

21. Wint G, Robinson T. Gridded livestock of the world. Food and Agriculture Organization of the United Nations, Rome; 2007.

22. Tatem AJ. WorldPop, open data for spatial demography. Scientific Data. 2017;4:170004.

23. Utazi C, Thorley J, Alegana V, Ferrari M, Nilsen K, Takahashi S, et al. A spatial regression model for the disaggregation of areal unit based data to high-resolution grids with application to vaccination coverage mapping. Statistical Methods in Medical Research. 2018; p. 096228021879736. doi:10.1177/0962280218797362.

24. Robinson TP, William Wint GR, Conchedda G, Van Boeckel TP, Ercoli V, Palamara E, et al. Mapping the global distribution of livestock. PLoS ONE. 2014;9(5):e96084. doi:10.1371/journal.pone.0096084.

25. Gilbert M, Nicolas G, Cinardi G, Van Boeckel TP, Vanwambeke SO, Wint GRWW, et al. Global distribution data for cattle, buffaloes, horses, sheep, goats, pigs, chickens and ducks in 2010. Scientific Data. 2018;5(1):1–11. doi:10.1038/sdata.2018.227.

26. Seré C, Steinfeld H. World livestock production systems-Current status. Issues and Trends (Food Agriculture Organization, Rome). 1996;doi:10.1002/pssc.201510263.

27. Gilbert M, Chaitaweesub P, Parakamawongsa T, Premashthira S, Tiensin T, Kalpravidh W, et al. Free-grazing ducks and highly pathogenic avian influenza, Thailand. Emerging infectious diseases. 2006;12(2):227–34. doi:10.3201/eid1202.050640.

28. Van Boeckel TP, Thanapongtharm W, Robinson T, D'Aietti L, Gilbert M. Predicting the distribution of intensive poultry farming in Thailand. Agriculture, Ecosystems & Environment. 2012;149:144–153. doi:10.1016/J.AGEE.2011.12.019.

29. Lawrence RL, Wood SD, Sheley RL. Mapping invasive plants using hyperspectral imagery and Breiman Cutler classifications (randomForest). Remote Sensing of Environment. 2006;100(3):356–362. doi:10.1016/J.RSE.2005.10.014.

30. Gislason PO, Benediktsson JA, Sveinsson JR. Random Forests for land cover classification. Pattern Recognition Letters. 2006;27(4):294–300. doi:10.1016/J.PATREC.2005.08.011.

31. Prosser DJ, Wu J, Ellis EC, Gale F, Van Boeckel TP, Wint W, et al. Modelling the distribution of chickens, ducks, and geese in China. Agriculture, Ecosystems and Environment. 2011;141(3-4):381–389. doi:10.1016/j.agee.2011.04.002.

32. OpenStreetMap Contributors. OpenStreetMap; 2014.

33. Center for International Earth Science Information Network (CIESIN) — Columbia University. Gridded population of the world, version 4 (GPWV4): population density; 2016.

34. IUCN, UNEP-WCMC. The World Database on Protected Areas (WDPA); 2010. Available from: `www.protectedplanet.net`.

35. Nelson A. Travel time to major cities: A global map of Accessibility. Ispra: European Commission. 2008;.

36. Weiss DJ, Nelson A, Gibson HS, Temperley W, Peedell S, Lieber A, et al. A global map of travel time to cities to assess inequalities in accessibility in 2015. Nature. 2018;553(7688):333–336. doi:10.1038/nature25181.

37. Land Process Distributed Active Archive Center (LDAAC). Global 30 Arc-Second Elevation Data Set GTOPO30; 2004.

38. Scharlemann JPW, Benz D, Hay SI, Purse BV, Tatem AJ, Wint GRW, et al. Global Data for Ecology and Epidemiology: A Novel Algorithm for Temporal Fourier Processing MODIS Data. PLoS ONE. 2008;3(1):e1408. doi:10.1371/journal.pone.0001408.

39. Jones P, Policy PTES&, undefined 2009. Croppers to livestock keepers: livelihood transitions to 2050 in Africa due to climate change. Elsevier;.

40. Zhang X, Friedl MA, Schaaf CB, Strahler AH, Hodges JCF, Gao F, et al. Monitoring vegetation phenology using MODIS. Remote Sensing of Environment. 2003;84(3):471–475. doi:10.1016/S0034-4257(02)00135-9.

41. Hansen MC, Potapov PV, Moore R, Hancher M, Turubanova S, Tyukavina A, et al. High-resolution global maps of 21st-century forest cover change. science. 2013;342(6160):850–853.

42. Arino O, Ramos Perez JJ, Kalogirou V, Bontemps S, Defourny P, Van Bogaert E. Global land cover map for 2009 (GlobCover 2009). ESA & UCL. 2012;.

43. Fick SE, Hijmans RJ. WorldClim 2: new 1-km spatial resolution climate surfaces for global land areas. International Journal of Climatology. 2017;37(12):4302–4315. doi:10.1002/joc.5086.

44. Balk D, Yetman G. The global distribution of population: evaluating the gains in resolution refinement. New York: Center for International Earth Science Information Network (CIESIN), Columbia University. 2004;.

45. Linard C, Gilbert M, Tatem AJ. Assessing the use of global land cover data for guiding large area population distribution modelling. GeoJournal. 2011;76(5):525–538. doi:10.1007/s10708-010-9364-8.

46. R Development Core Team R. R: A Language and Environment for Statistical Computing; 2011. Available from: `http://www.r-project.org`.

47. Swift A, Liu L, Uber J. Reducing MAUP bias of correlation statistics between water quality and GI illness. Computers, Environment and Urban Systems. 2008;32(2):134–148. doi:10.1016/j.compenvurbsys.2008.01.002.

48. Tuson M, Yap M, Kok MR, Murray K, Turlach B, Whyatt D. Incorporating geography into a new generalized theoretical and statistical framework addressing the modifiable areal unit problem. International Journal of Health Geographics. 2019;18(1):1–15. doi:10.1186/s12942-019-0170-3.

49. Fox J. People and the environment : approaches for linking household and community surveys to remote sensing and GIS. Kluwer Academic Publishers; 2003.

50. Cebrecos A, Domínguez-Berjón MF, Duque I, Franco M, Escobar F. Geographic and statistic stability of deprivation aggregated measures at different spatial units in health research. Applied Geography. 2018;95:9–18. doi:10.1016/J.APGEOG.2018.04.001.

51. Rohde D, Corcoran J, Chhetri P. Spatial forecasting of residential urban fires: A Bayesian approach. Computers, Environment and Urban Systems. 2010;34(1):58–69. doi:10.1016/J.COMPENVURBSYS.2009.09.001.

52. Xu P, Huang H, Dong N, Abdel-Aty M. Sensitivity analysis in the context of regional safety modeling: Identifying and assessing the modifiable areal unit problem. Accident Analysis & Prevention. 2014;70:110–120. doi:10.1016/J.AAP.2014.02.012.

53. Truong PN, Stein A. A hierarchically adaptable spatial regression model to link aggregated health data and environmental data. Spatial Statistics. 2018;23:36–51. doi:10.1016/J.SPASTA.2017.11.002.

54. Tagashira N, Okabe A. The Modifiable Areal Unit Problem, in a Regression Model Whose Independent Variable Is a Distance from a Predetermined Point. Geographical Analysis. 2002;34(1):1–20. doi:10.1111/j.1538-4632.2002.tb01072.x.

55. Parenteau MP, Sawada MC. The modifiable areal unit problem (MAUP) in the relationship between exposure to NO 2 and respiratory health. International journal of health geographics. 2011;10(1):58.

56. Mitra R, Buliung RN. Built environment correlates of active school transportation: neighborhood and the modifiable areal unit problem. Journal of Transport Geography. 2012;20(1):51–61. doi:10.1016/J.JTRANGEO.2011.07.009.

57. Lee G, Cho D, Kim K. The modifiable areal unit problem in hedonic house-price models. Urban Geography. 2016;37(2):223–245. doi:10.1080/02723638.2015.1057397.

58. Goodman AC, et al. A comparison of block group and census tract data in a hedonic housing price model. Land Economics. 1977;53(4):483–487.

59. Wakefield J. Sensitivity Analyses for Ecological Regression. Biometrics. 2003;59(1):9–17. doi:10.1111/1541-0420.00002.

Figure 1

Click here to access/download;Figure;Fig1.TIF

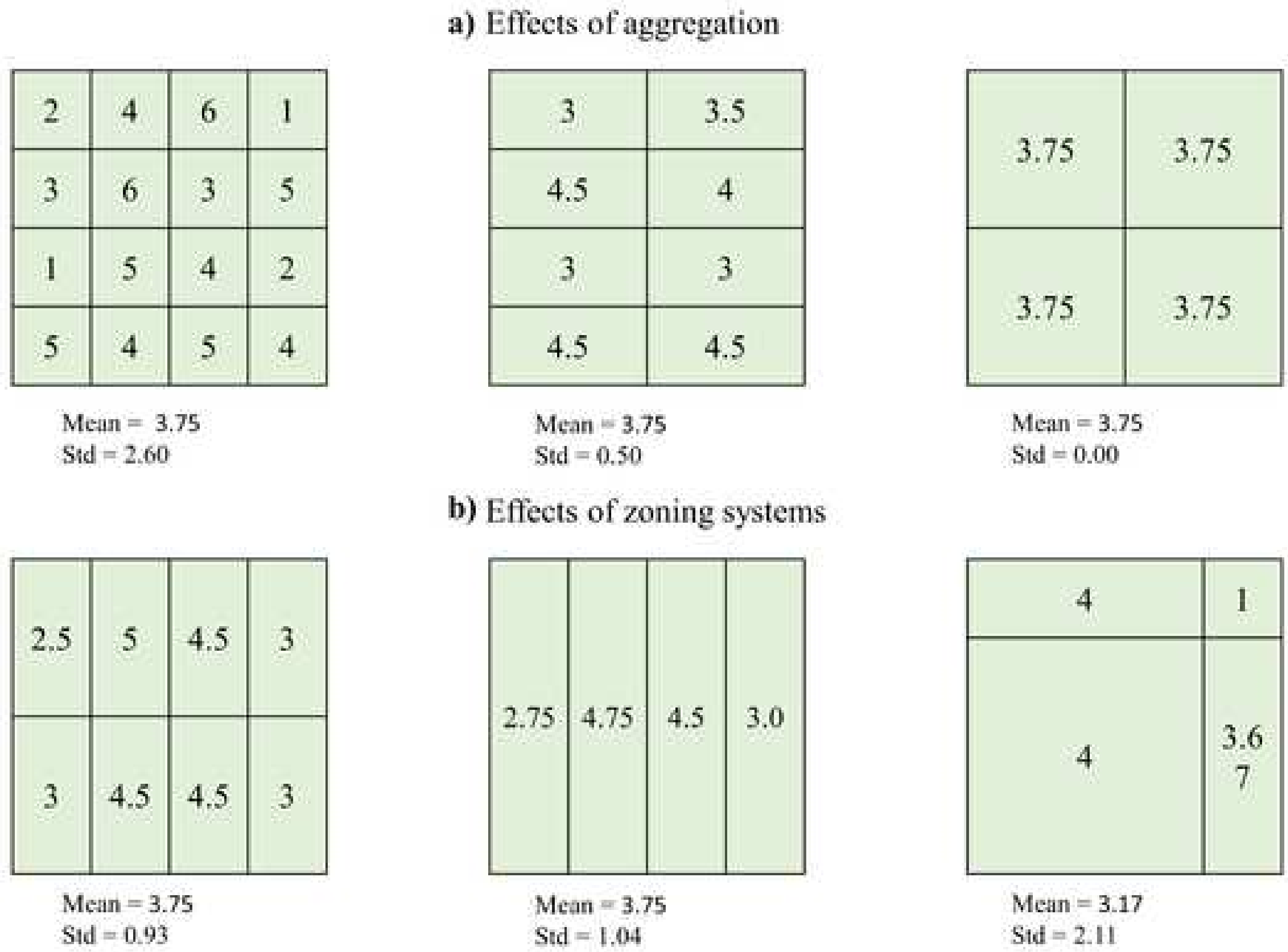

Figure 2

Click here to access/download;Figure;Fig2.TIF

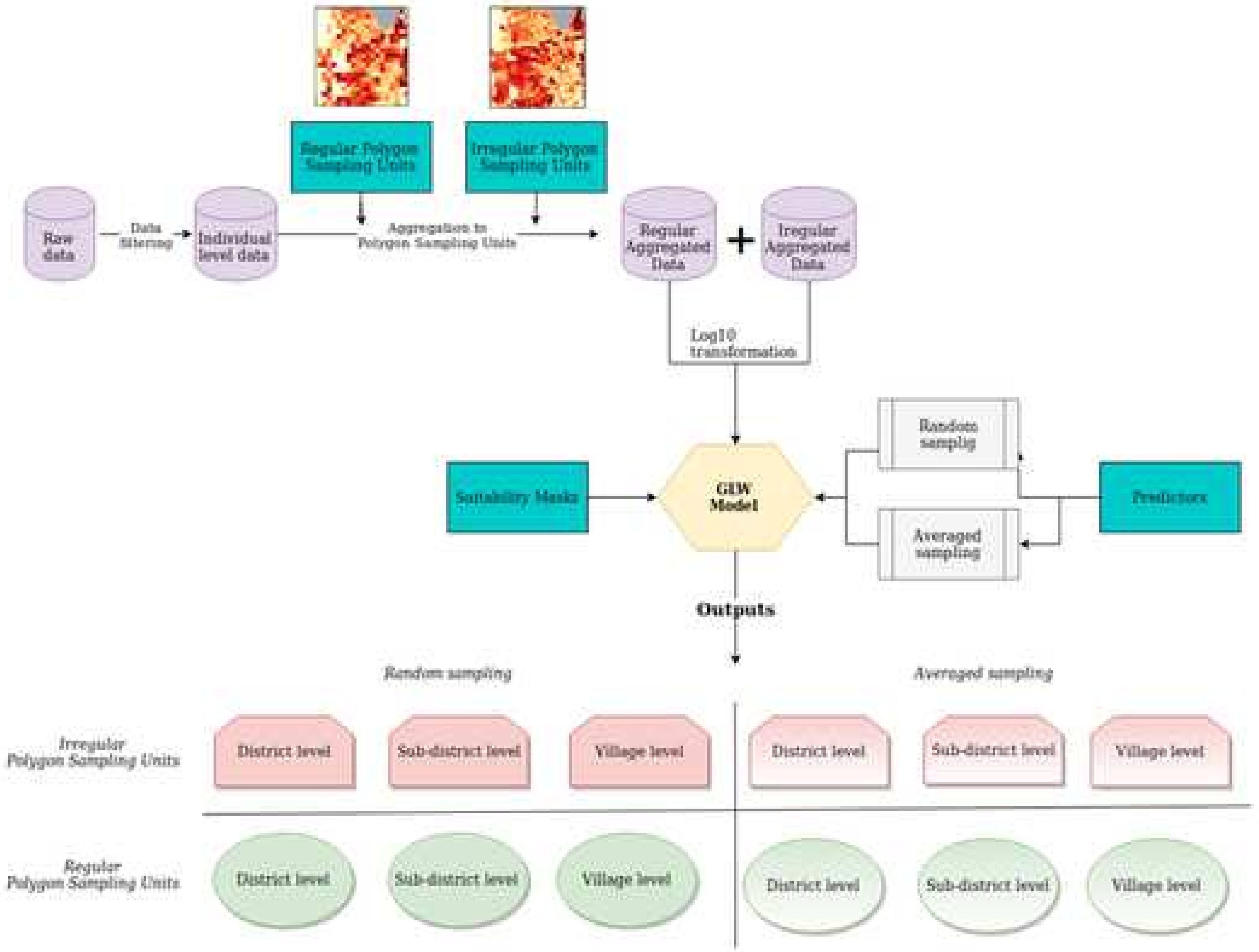

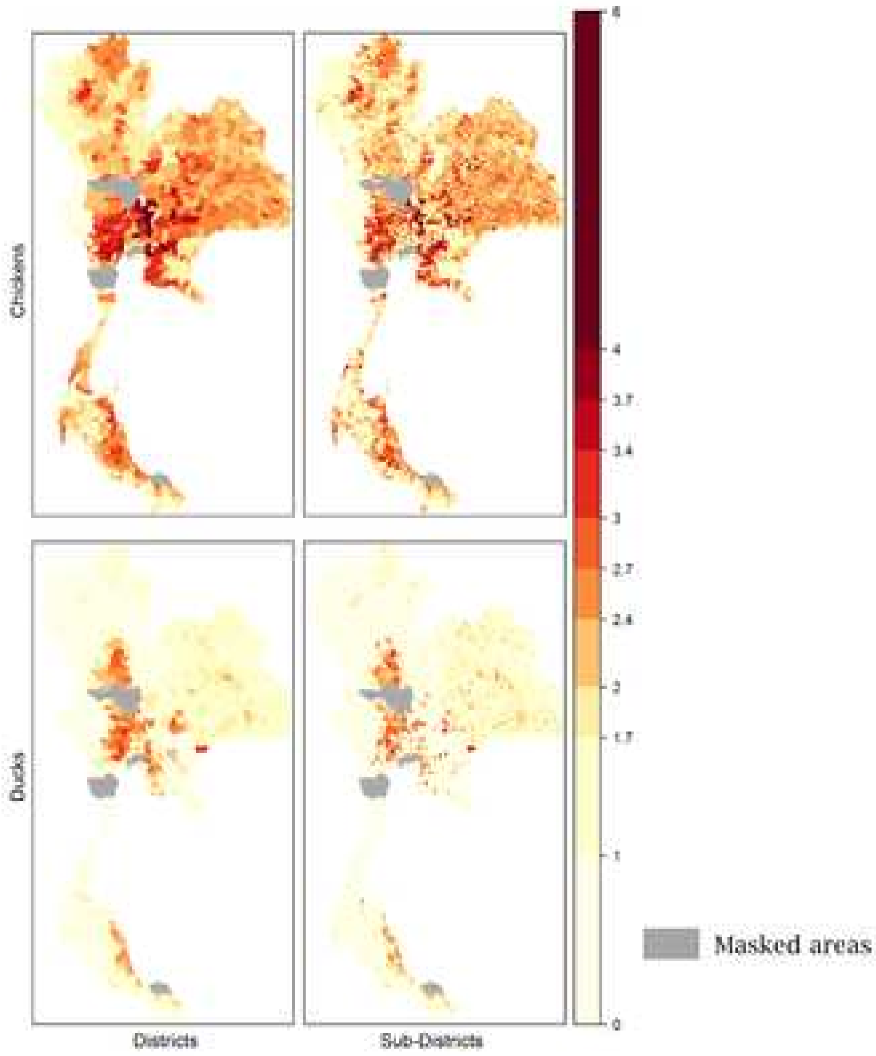
Chickens
Ducks
Districts
Sub-Districts
4
3.7
3.4
3
2.7
2.4
2
1.7
1
0
Masked areas

Figure 4

Click here to access/download;Figure;Fig4.TIF

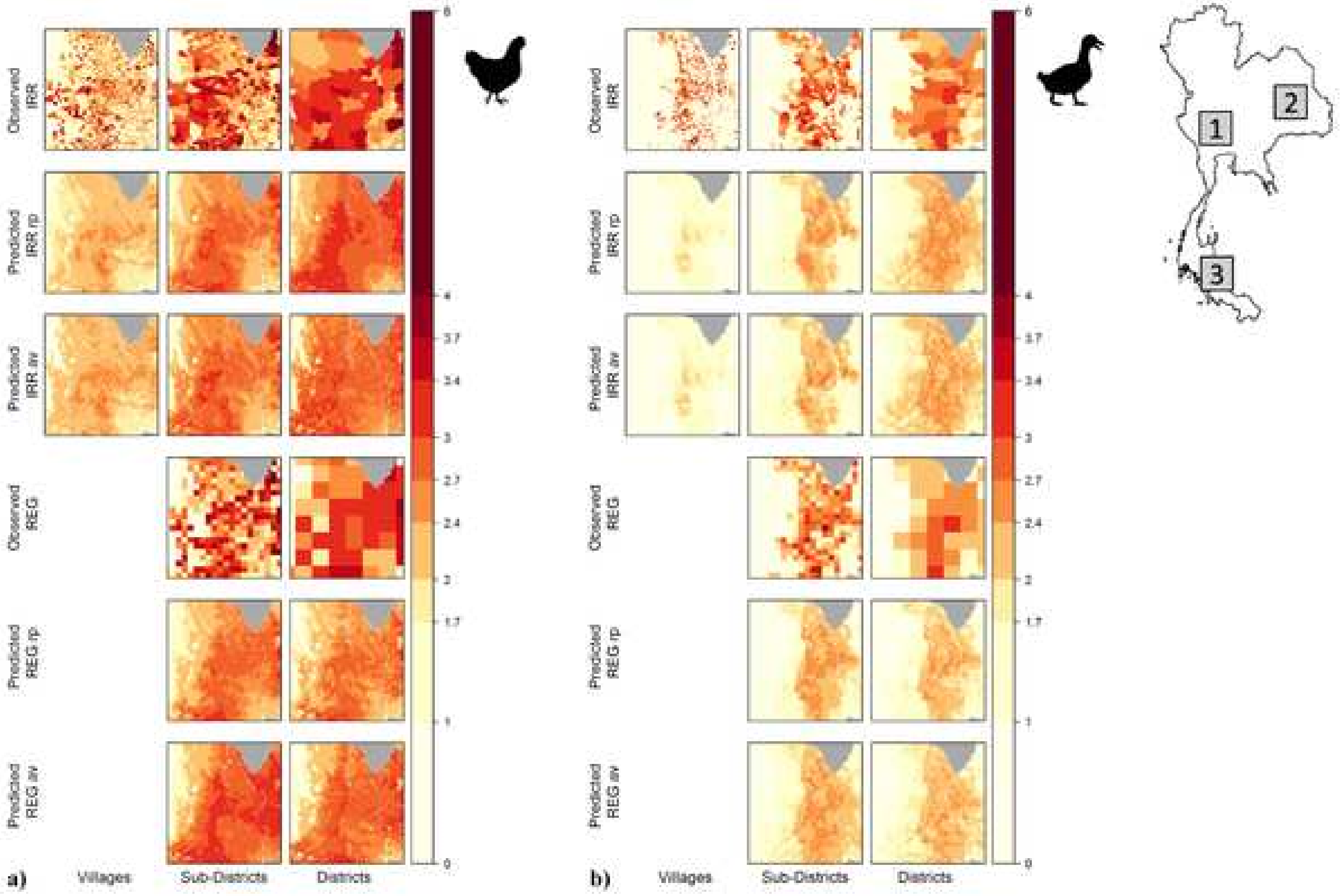

Figure 5

Click here to access/download;Figure;Fig5.TIF

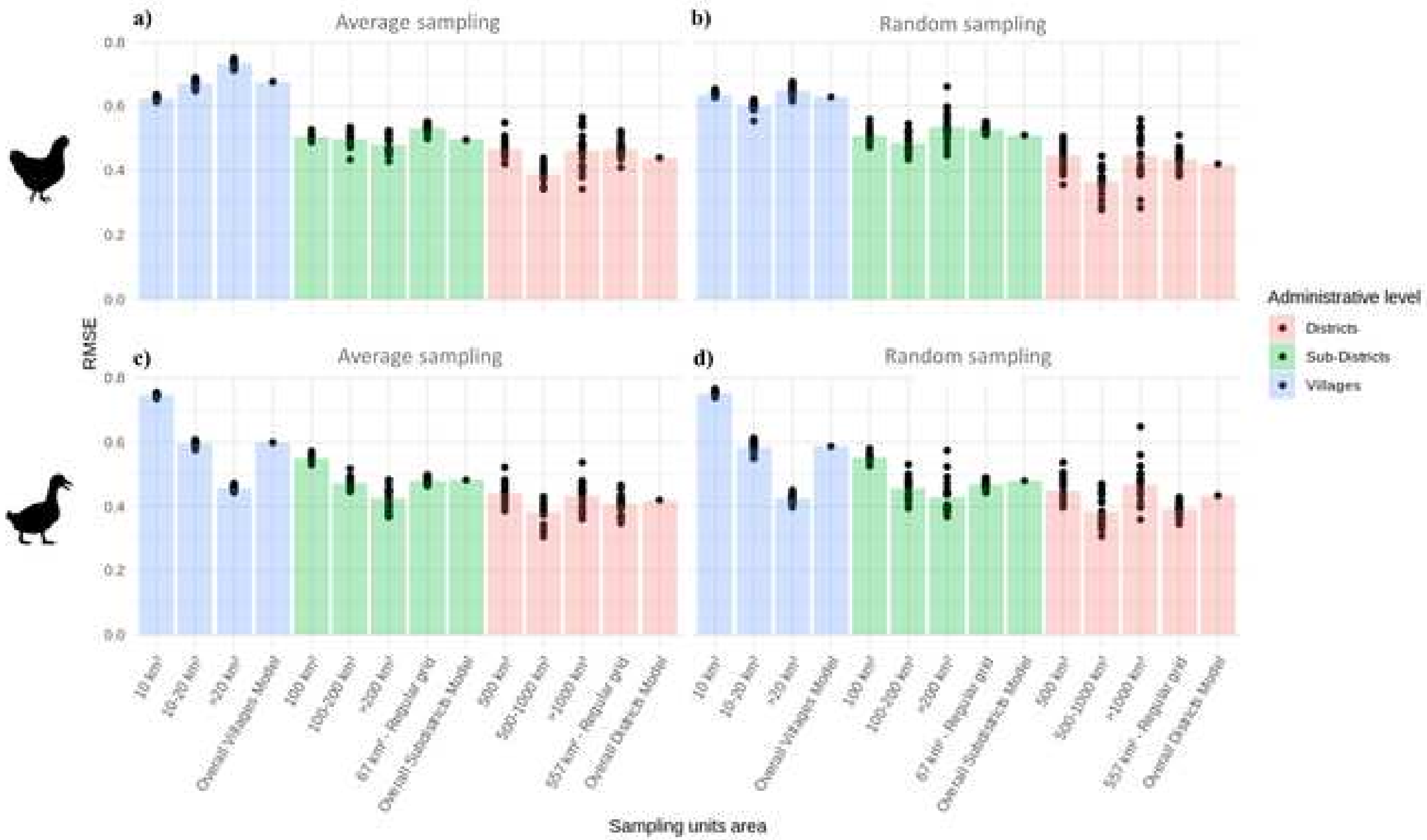

Figure 6

Click here to access/download;Figure;Fig6.TIF

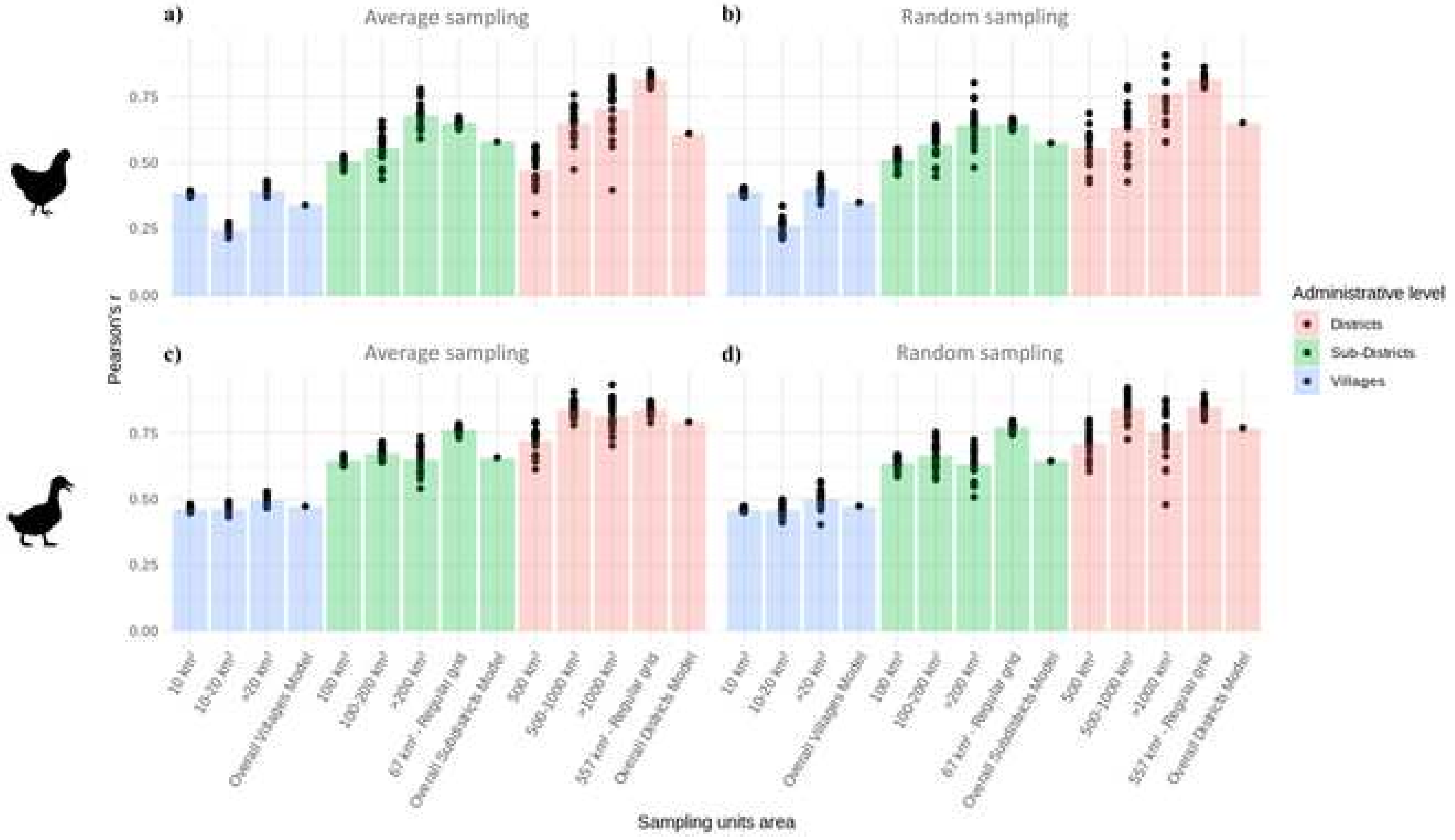

Figure 7

Click here to access/download;Figure;Fig7.TIF

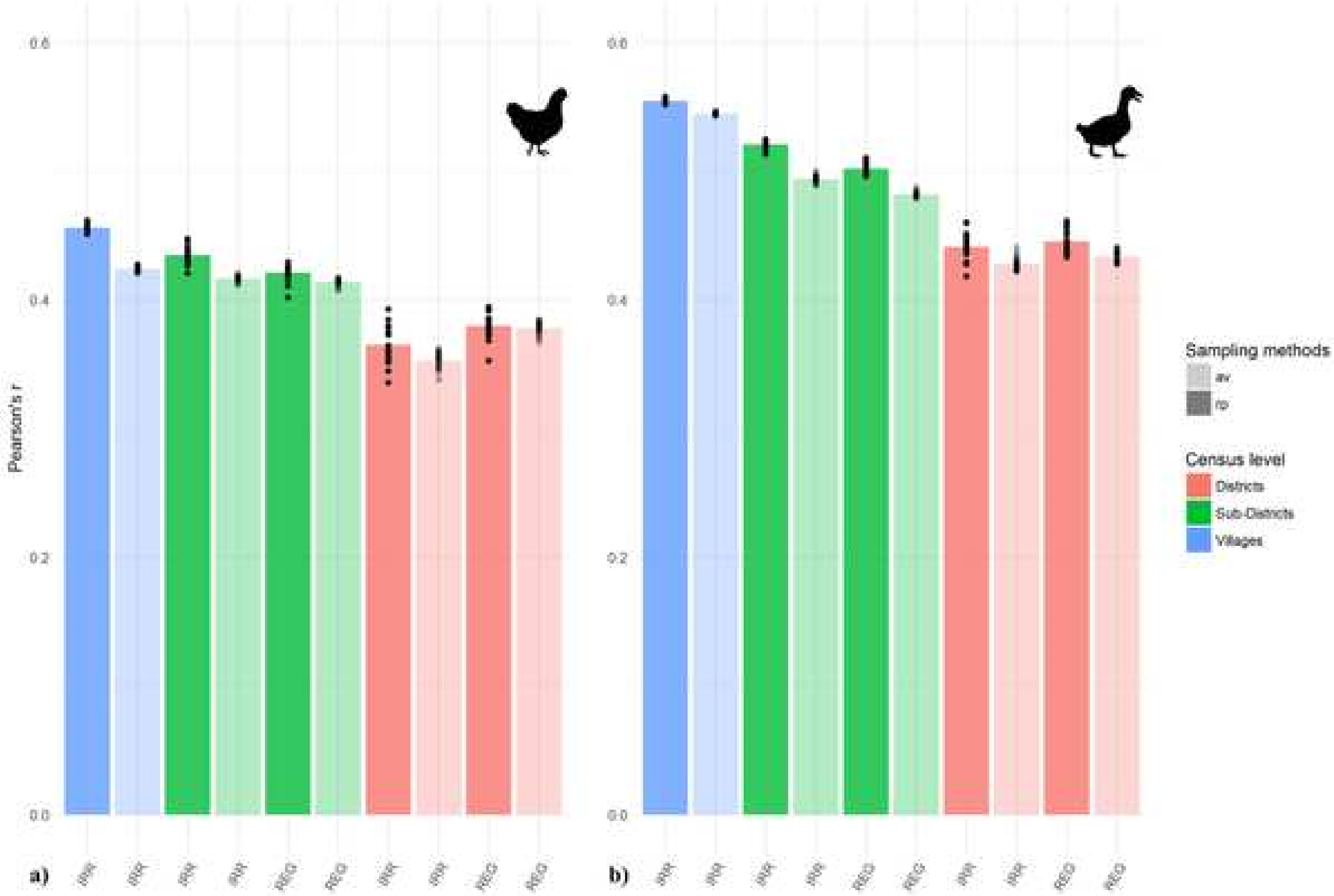

SI tab 1

Click here to access/download
Supporting Information
s1_table.csv

SI tab 1

Click here to access/download
Supporting Information
s1_table.tex

raw images

Click here to access/download
Supporting Information
S1_raw_images.pdf

Click here to access/download
Supporting Information
S1_raw_supporting_info.pdf

Supporting Information Fig 1

Click here to access/download
Supporting Information
S1.TIF

Supporting Information Fig 3

Click here to access/download
Supporting Information
S3.TIF

Supporting Information Fig 4

Click here to access/download
Supporting Information
S4.TIF

Supporting Information Fig 2.1

Click here to access/download
Supporting Information
S21.TIF

Supporting Information Fig 2.2

Click here to access/download
Supporting Information
S22.TIF

Supporting Information Fig 3.1

Click here to access/download
Supporting Information
S31.TIF

Supporting Information Fig 3.2

Click here to access/download

Supporting Information

S32.TIF

Supporting Information Fig 4.1

Click here to access/download
Supporting Information
S41.TIF

Supporting Information Fig 4.2

Click here to access/download
Supporting Information
S42.TIF
