# Supplementary material for: Downscaling livestock census data using multivariate predictive models: Sensitivity to modifiable areal unit problem
Source: PLoS One. 2020 Jan 27;15(1):e0221070. doi: 10.1371/journal.pone.0221070 (PMC6984718; doi:10.1371/journal.pone.0221070)
Supplement: Attachment — Submitted filename: PONE-D-19-20969_RebuttalLetter_Reply2reviewers.pdf [file pone.0221070.s012.pdf]

Louvain-la-neuve, 27th November 2019

Sotirios Koukoulas, PhD
Academic Editor
PLOS ONE

Re: Submission to PLOS ONE– Revised Manuscript

Dear Dr Koukoulas,

We would like to thank you and the referees for the time and efforts dedicated to our submission entitled “Downscaling livestock census data using multivariate predictive models: sensitivity to modifiable areal unit problem” (PONE-D-19-20969) by Daniele Da Re, Marius Gilbert, Celia Chaiban, Pierre Bourguignon, Weerapong Thanapongtharm, Timothy P. Robinson and Sophie O. Vanwambeke. We attach to this letter the revised version of our manuscript.

We have carefully revised the manuscript to address all of the reviewers’ comments. Below we describe a point-by-point response to the comments provided by the referees. We believe that the new version of the manuscript is clearer and stronger. We deeply thank the anonymous reviewers for the constructive and positive suggestions.

Please do not hesitate in contacting me if I can be of further assistance.

On behalf of all co-authors,

Daniele Da Re

Reviewers' Comments and Authors Response

Reviewer 1

Please use the space provided to explain your answers to the questions above. You may also include additional comments for the author, including concerns about dual publication, research ethics, or publication ethics. (Please upload your review as an attachment if it exceeds 20,000 characters)

Reviewer #1: This paper addresses the MAUP, an important issue in spatial analysis that is related to changing spatial scale. Although the paper is well written, I recommend the authors adding clarifications for several elements as follows:

1. Statement in lines 29-31 seems not telling the complete review on the research of MAUP. There are many published studies on the MAUP issues for downscaling or spatial disaggregation.

A (Authors' response): We have edited the sentence highlighting the fact that, as far as we know, the MAUP issue was more addressed in the aggregation and in relation to the changes of models estimates rather than investigating the effect of MAUP in the dowscaling precision.

2. Explain what the individual level data at line 77-78 are? How many of them? For the whole country or not? In which year? Etc., a detailed description of the data at individua level is required.
A: We specified at L78 that the individual level data are the poultry census described in "Poultry population data" section.

3. Line 76: which aggregation method was used, linear or non-linear aggregation?
A: We used simple additive aggregation method, we have now specified it at L78 of the revised manuscript.

4. Line 90-99: explain why those predictors were used with reference.
A: We have specified it at L93-94.

5. Line 97: explain why log-transform was applied to poultry density? What correction was used as mentioned in line 97 (poultry densities corrected by area).
A: For the sake of brevity, and given the general use of these setting in the GLW framework, we refer the interested reader to the paper by Gilbert et al 2018 were this is fully explained.

6. Provide a map that shows the three administration units in Thailand: village, sub-district and district, how large are they relatively to each other?
A: We have not provided a map showing the three administrative units because at country scale it would not have been possible to appreciate the differences among them. However, we have specified the Average Spatial Resolution (ASR) of the three administrative units at L114-115.

7. Also provide the maps visualizing IRR and REG PSUs. Are the IRR PSUs the same as the three administration units above?
A: IRR and REG PSUs can be observed in Fig3. The IRR PSUs do correspond to the three administration units described above.

8. What are the census polygons in line 118?
A: The polygons used to train the model. We have called them *polygons* now.

9. Fig 2 has poor quality, I cannot read them all.
A: We have improved the resolution of Fig2.

10. Why are there two separate sections for model evaluation and downscaling precision? Are they not the same? What is the difference between COR in line 179 and CORdown in line 187?
A: As specified at L42, CORdown "(...) Pearson's *r* was computed between predictions and the observed data at the village level only to assess the capacity of models trained using various PSUs to predict poultry population at a fine scale".

11. Overall, I find the findings of this paper obvious. Should this be again published the facts that we all know?
A: Though obvious, we believe that our sensitivity analysis and findings are needed to support the increase use of the GLW and other downscaled data products, in order to make the users aware of the potential issue of this data product itself and of all the downscaled data product that are becoming more and more available every year.

Reviewer 2

12. My review has turned out to be more of a proof reading session than anything else I have up;loaded an annotated pdf with quite a few very minor linguistic changes highlighted.
A: We thank Reviewer2 for his/her positive comment. All the comments in the PDF have been addressed.

13. The science seems clean and well presented , and pretty much acceptable as it is. I have only one minor technical question - namely about the use of Voronoi polygons around villages - doesn't this mean that any density calculated for the polygon is affected by the distance between villages. I dont think this affects the validity of the study, but might it affect the values of the means calculated and this the comparison between different scales??.

In addiction Comment at L78: *Doest using Voronoi polygon then mean the the animals are spread throughout the polygon...i.e the training density depends on the size of the polygons - or the distance between villages? Might it have been better to use a fixed buffer or grid instead so the poultry density wasnt affected by village density??*

A: We compute the Voronoi polygons because villages administrative units for Thailand are not available. This was a simple methodological choice in order to get small scale polygons and have the chance to train the GLW model using different polygons scales.

14. Not a major problem though, so I am happy to recommend acceptance with very minor revisions
A: We thank Reviewer2 for his/her positive assessment.

15. Comment on L218: ??coarse predicitions despite the fact that the predictors were the same resolution (500m) for all models
A: We are referring to the scale of the training polygons dataset and not to the scale and spatial resolution of the predictors. We have specified in the revised version that we are referring to the scale of the training polygons.

16. Comment on L218: Accordingly:In agreement with ??BUT SEE your line 220 re MAUP scale effect- coarser training data gave better accuracy. Is this not a contradiction?
or have i missed the point??
A: The dowscaling precision (CORdown) measures the "(...) Pearson's *r* (…) between predictions and the observed data at the village level only to assess the capacity of models trained using various PSUs to predict poultry population at a fine scale". It is a measure of the performances of the GLW model to downscale a polygon, which is better when the training polygon is small.

On the contrary, if we want to asses the precision of the model when predicting the whole response variable distribution, it provides better estimates when the model is trained with coarser polygons, due to the narrowing effect of the data distribution caused by the MAUP-scale effect (please see e.g. Supplementary Materials 2.1).
